# Dual Blockade of EP2 and EP4 Signaling is Required for Optimal Immune Activation and Antitumor Activity Against Prostaglandin-Expressing Tumors

**DOI:** 10.1158/2767-9764.CRC-23-0249

**Published:** 2023-08-08

**Authors:** Brian J. Francica, Anja Holtz, Justine Lopez, David Freund, Austin Chen, Dingzhi Wang, David Powell, Franciele Kipper, Dipak Panigrahy, Raymond N. Dubois, Chan C. Whiting, Peppi Prasit, Thomas W. Dubensky

**Affiliations:** 1 Tempest Therapeutics, Brisbane, California.; 2 Department of Biochemistry and Molecular Biology, Hollings Cancer Center, Medical University of South Carolina, Charleston, South Carolina.; 3 Department of Pathology, Beth Israel Deaconess Medical Center and Harvard Medical School, Boston, Massachusetts.

## Abstract

While the role of prostaglandin E2 (PGE2) in promoting malignant progression is well established, how to optimally block the activity of PGE2 signaling remains to be demonstrated. Clinical trials with prostaglandin pathway targeted agents have shown activity but without sufficient significance or dose-limiting toxicities that have prevented approval. PGE2 signals through four receptors (EP1–4) to modulate tumor progression. EP2 and EP4 signaling exacerbates tumor pathology and is immunosuppressive through potentiating cAMP production. EP1 and EP3 signaling has the opposite effect through increasing IP3 and decreasing cAMP. Using available small-molecule antagonists of single EP receptors, the cyclooxygenase-2 (COX-2) inhibitor celecoxib, or a novel dual EP2/EP4 antagonist generated in this investigation, we tested which approach to block PGE2 signaling optimally restored immunologic activity in mouse and human immune cells and antitumor activity in syngeneic, spontaneous, and xenograft tumor models. We found that dual antagonism of EP2 and EP4 together significantly enhanced the activation of PGE2-suppressed mouse and human monocytes and CD8^+^ T cells *in vitro* as compared with single EP antagonists. CD8^+^ T-cell activation was dampened by single EP1 and EP3 antagonists. Dual EP2/EP4 PGE2 receptor antagonists increased tumor microenvironment lymphocyte infiltration and significantly reduced disease burden in multiple tumor models, including in the adenomatous polyposis coli (APC)^min+/−^ spontaneous colorectal tumor model, compared with celecoxib. These results support a hypothesis that redundancy of EP2 and EP4 receptor signaling necessitates a therapeutic strategy of dual blockade of EP2 and EP4. Here we describe TPST-1495, a first-in-class orally available small-molecule dual EP2/EP4 antagonist.

**Significance:**

Prostaglandin (PGE2) drives tumor progression but the pathway has not been effectively drugged. We demonstrate significantly enhanced immunologic potency and antitumor activity through blockade of EP2 and EP4 PGE2 receptor signaling together with a single molecule.

## Introduction

Malignant progression is associated with induced cyclooxygenase-2 (COX-2) expression and increased synthesis of prostaglandin E2 (PGE2) from arachidonic acid intermediates (1, 2). PGE2 signals through four consecutively named homologous cell surface receptors, EP1–4, that are differentially expressed depending on cell lineage and environmental cues (3). The intracellular signal delivered in response to binding of PGE2 to each of its four targeted EP receptors depends on the alpha subunit of the E-prostanoid G protein–coupled receptor and informs the development of new therapeutics which selectively target the prostaglandin pathway (4, 5). In general, EP1 and EP3 signaling potentiates immunity by increasing calcium flux and decreasing cAMP levels (6, 7). In contrast, EP2 and EP4 signaling suppresses immunity by stimulating cAMP production and downregulating T-cell and myeloid cell activation by signaling through protein kinase A (8, 9).

PGE2 promotes tumorigenesis, tumor cell survival and metastasis, angiogenesis, and fibroblast development in the tumor microenvironment (TME) through both autocrine and paracrine signaling (2, 10–15). Tumor- or stroma-produced PGE2 is profoundly immune suppressive and facilitates evasion of immune recognition by inhibiting the effector functions of myeloid cell and lymphocyte populations and enhancing the function of suppressive immune cell populations (16–19). Elevated COX-2 levels are associated with tumor progression in both human cancers and animal tumor models and have diverse etiologies including inflammation, tumor-driver mutations, radiation, and chemotherapy-induced immunogenic cell death (2, 20–23). In addition, recent investigations have suggested that elevated COX-2 production and expression of EP2 and EP4 in tumor cells may be associated with adaptive immune resistance to immune checkpoint inhibitor therapies (24–26).

Despite the well-established linkage between PGE2 production and tumor progression, there are no FDA-approved cancer therapeutics targeting the prostaglandin pathway. Broad inhibition of PGE2 signaling pathways with NSAIDs that block both COX-1 and COX-2 (e.g., naproxen), or those that block only COX-2 (e.g., celecoxib), results in antitumor effects in mouse syngeneic tumor models. Epidemiologic and prospective studies indicate that inhibition of the PGE2 pathway can translate to clinical benefit (27–29). Indeed, regular use of NSAIDs after diagnosis with colorectal cancer and head and neck cancer has been associated with improved outcomes in PIK3CA-mutated cancers (30, 31). However, gastrointestinal, renal, and cardiac toxicities are associated with chronic usage of COX inhibitors due to imbalances in prostacyclin and thromboxane levels limiting both tolerable dose levels and their long-term use for treatment of advanced cancers.

Here we describe the *in vitro* and *in vivo* activity of TPST-1495, a first-in-class dual EP2/EP4 PGE2 receptor antagonist. TPST-1495 is an orally bioavailable small molecule with potent and selective activity against the EP2 and EP4 receptors while sparing the structurally homologous, yet differentially active, EP1 and EP3 counterparts. We show that TPST-1495 was significantly more potent than single EP receptor antagonists or COX-2 inhibition at inducing immune activation, antitumor immunity, and tumor clearance in multiple models of human malignancies, highlighting the redundancy of EP2 and EP4 signaling in the tumor and the necessity of dual receptor blockade. These results support the scientific rationale for a new cancer therapeutic approach based on the targeted combined antagonism of EP2 and EP4.

## Materials and Methods

### TPST-1495 Selection and IC_50_ Determination

Cell lines stably expressing EP1, 2, 3, or 4 receptors were purchased from Eurofins Panlabs, Inc (catalog nos. HTS142L, HTS185L, HTS099C, and HTS092L). EP2 and EP4 Cell lines were cultured in T-150 media for up to 50 passages. Cells were passaged every 3–5 days when less than 80% confluent. EP1 and EP3 cell lines were thawed immediately prior to use per manufacturer's recommendations. To facilitate calcium flux assay readout, cloned EP2 and EP4 receptor-expressing ChemiBrite cells were made by stable transfection of HEK293 cells with ChemiBrite clytin, EP2 or EP4 receptor linked genetically to a promiscuous G protein to couple EP receptors to the calcium signaling pathway. Cells were plated 18–24 hours prior to assay initiation in basal media per manufacturer's recommendations. For EP2 and EP4 stable cell lines, 2 × 10^4^ cells were plated per well. For EP1 and EP3, cells were thawed and plated into two 96-well plates. Hank's Balanced Salt Solution (HBSS) + 20 mmol/L HEPES pH 7.4 was used as dilution assay buffer. A total of 10 μmol/L Coelenterazine (Invitrogen) assay buffer was used as loading buffer and prepared the day of conducting the assay. Plates were incubated in dark for 2–3 hours. PGE2 (Sigma P5640-5 mg) was used at a final concentration of 10 nmol/L for EP1, EP2, and EP4 assay. For EP3 assay, PGE2 was used at a final concentration of 200 nmol/L. Plates were incubated in dark for 2–3 hours. Serial 11-point half log dilutions of TPST-1495 were prepared beginning at 30 μmol/L final concentration for EP1 and EP3 and 1 μmol/L for EP2 and EP4. Antagonist was added at 25 μL per well and incubated for 20 minutes prior to being read on a Flexstation (Molecular Devices). TPST-1495 wells were run in duplicate. Control wells were run in quadruplicate.

### PGE2 Blockade Whole Blood Assay

The PGE2 Blockade whole blood assay was performed using mouse and human whole blood. Human whole blood assays were performed with fresh, same day drawn healthy donor whole blood (catalog no. 70507.2, StemCell Technologies) and plated at 75 μL per well. Mouse whole blood assays were performed with whole blood drawn from terminal cardiac puncture of up to 15 female Balb/c mice and plated at 75 μL per well. Whole blood was then treated with dilutions of TPST-1495 and incubated for 30 minutes at 37°C, followed by 10 nmol/L or 500 nmol/L PGE2 (catalog no. P0409, Sigma-Aldrich) and incubated for 30 minutes at 37°C. Lipopolysaccharide (LPS) was then added to a final concentration of 0.5 mg/mL and the combination of these reagents was incubated at 37°C for 16–20 hours, at which point plates were isolated to separate supernatant for collection. TNFα was measured by murine (catalog no. BMS6073, Thermo Fisher Scientific) or human (catalog no. BMS223, Thermo Fisher Scientific) ELISA.

### PGE2 Blockade Assay with T-cell Activation

Human T-cell assays were performed with peripheral blood mononuclear cell (PBMC) isolated from a fresh leukopak (catalog no. 70500.2, StemCell Technologies). Human PBMC were thawed in warm RPMI+ Benzonase, enriched for T cells using magnetic separation (catalog no. 130-096-535, Miltenyi Biotec), then plated at 2 × 10^6^ cells/mL in 100 μL RPMI supplemented with 10% FBS (catalog no. 10438026, Gibco) and penicillin-streptomycin (catalog no. 15140163, Gibco). Mouse T-cell activation assays were performed with unenriched single-cell suspensions isolated after red blood cell (RBC) lysis (catalog no. A1049201, Gibco) of pooled lymph nodes and spleens from OT-1 Mice [Jax, C57BL/6-Tg(TcraTcrb)1100 Mjb/J] and plated at 0.5–1 × 10^6^ cells per well. In both human and mouse assays, cells were then preincubated with EP inhibitors for 20 minutes at 37°C, followed by PGE2 (catalog no. P0409, Sigma-Aldrich) for 20 minutes at 37°C, exposed to varying concentrations of PGE2 (10–1,000 nmol/L) for 30 minutes at 37°C. After these preincubation steps, CEF peptides (catalog no. 3616-1, Mabtech) or SIINFEKL peptide (catalog no. S7951, Sigma-Aldrich) were added, and all components were incubated for 6 additional hours at 37°C. At that time, supernatants were collected, and cytokines were measured by Luminex (catalog no. HCYTA-60K-PX48, Millipore).

### Cell Culture

CT26, B16.F10, Lewis lung carcinoma (LLC), and 4T1 cells were acquired from ATCC (Product: CRL-2638, CRL-6475, CRL-1642, and CRL-2539), grown in RPMI (Gibco A1049101) supplemented with 10% FBS (Gibco 10438026). TS/A cells were a generous gift from Dr. Dipak Panigrahy and were grown in RPMI supplemented with 10% FBS. All cells were grown at 37°C in the presence of 5% CO_2_. According to established Tempest “Work Instruction” protocols, all mouse cell lines were verified by ATCC using their mouse cell authentication short tandem repeat (STR) profiling (ATCC 30-1012K). Cells were considered a match if at least 17 STR loci matched the published information. In addition, cell lines were screened for *Mycoplasma* using ATCC's universal *Mycoplasma* detection kit (31-101K) when freezing. Cells were cultured for a maximum of 1 month and/or 8 passages from frozen cell stock lots stored under liquid nitrogen established at ≤10 passages.

### Animal Studies

All animal experiments were performed in accordance with the NIH Guidelines for the ethical care and use of laboratory animals and were approved by the Institutional Animal Care and Use Committee (IACUC) at the site of experimentation.

C57/BL6 (catalog no. 4404F, Envigo), APC^min/+^ (catalog no. 002020, Jax), RAG2^−/−^ BALB/c (catalog no. 601-F, Taconic), and Balb/c (catalog no. BALB-F, Taconic; catalog no. 4704F, Envigo) were housed in accordance with IACUC protocols and experimentation was performed at Murigenics, Crown Bio, or at our collaborator's institutions, the Beth Israel Deaconess Medical Center and Harvard Medical School, Boston, MA, or the Medical University of the South, Charleston, SC. Mice were allowed to acclimate to housing for >1 week before experiments began. For syngeneic allograft experiments (CT26, 4T1, LLC, TS/A, B16F10), tumor cells were implanted at 1 × 10^6^ cells in the flanks of 6–8 weeks old female Balb/c or C57/BL6 mice and allowed to grow to a group average of 80–100 mm^3^ and mice were then randomized and treatment was initiated. For CD8α depletion studies, CD8α depletion antibody (catalog no. BP0061, BioXCell) was administered on days −2 and −2 relative to TPST-1495 treatment and weekly thereafter. ASGM1 depletion was performed by administering 10 μL of Ultra-Leaf Purified anti- Asialo-GM1 antibody (catalog no. 146002, BioLegend) on days −2 and −1 relative to TPST-1495 treatment and weekly thereafter.

For the LS-174T xenograft model, 2.5 × 10^4^ LS-174T cells were injected into the cecal wall of 8-week-old male NSG mice. 5 days after injection, the mice bearing LS-174T cells were randomly divided into two groups treated with methylcellulose or methylcellulose containing TPST-1495 (25 mg/kg, twice a day) by gavage for 8 weeks.

For APC^min/+^*in vivo* tumor experiments, mice were obtained from JAX at 6–8 weeks and aged in-house until they were 12–13 weeks old. Mice were then administered a regimen of 100 mg/kg TPST-1495 orally every day or twice a day, E7046 (MedChem Express, HY-103088), PF-04419848 (SelleckChem, catalog no. S7211), or celecoxib (MedChem Express, Hy-14398) for 3 weeks, at which time tumors were resected and prepared for histology and RNA sequencing. In some experiments, mice were administered drugs for 6 weeks then followed for humane endpoints (lethargy, body weight) were reached for survival analysis, as described in the relevant Figure legends.

The dose, route, and regimen of celecoxib used for our studies was based on literature describing the activity and toxicity of COX inhibitors *in vivo*. The 60 mg/kg maximum dose used was well tolerated without cage-side observations and was the maximally effective dose for *in vivo* studies (32).

### Prostaglandin Pathway Inhibitor Drugs

PGE2 pathway inhibitors used are listed in Table 1A. Each inhibitor was resuspended in DMSO (catalog no. BP231-100, Thermo Fisher Scientific) at 50 mmol/L and kept frozen at −20°C until use.

**TABLE 1A:** Prostaglandin pathway inhibitor drugs

Target	Vendor	Catalog no.
EP1	Tocris	5406/50
EP2	Selleck Chemical	S7211
EP3	Sigma	L4545-25MG
EP4	Medchem Express	HY-103088
Celecoxib (COX-2 inhib.)	MedChemExpress	HY-14398

### Flow Cytometry

Tumors were processed for flow cytometry by maceration with a scissors followed by dissociation in Collagenase type IV (catalog no. LS004189, Worthington Biochemical Corporation) and Benzonase (catalog no. MFCD00131010, Sigma-Aldrich). No gradient separation was performed. Spleens and lymph nodes processed by mashing, straining in a 30–40 μm filter, and washing in PBS. Splenic RBCs were lysed with Ammonium-Chloride-Potassium (ACK) lysis buffer (catalog no. A1049201, Gibco). Blood PBMC were isolated using a density gradient separation with Lympholyte M (catalog no. CL5031, Cedarlane Labs) at 1,200 × *g* for 15 minutes. *Ex vivo* tumor-infiltrating lymphocyte (TIL) stimulation was performed by taking 10% of each tumor single-cell suspension and incubating according to the suggested dilutions of the Cell Stimulation Cocktail (catalog no. 00-4970-93, eBioscience) for 5 hours at 37°C, then overnight at 4°C. The cells were then stained for cell surface molecules and intracellular cytokines using the manufacturer protocol for the BD Biosciences Fixation/Permeabilization kit (catalog no. 554714, BD Biosciences). *Ex vivo* stimulation was performed by creating a single-cell suspension from tumors and stimulating with 1x cell stimulation cocktail (catalog no. 00-4970-93, eBioscience) for 5 hours in the presence or absence of Golgi Stop (catalog no. 554724, BD Biosciences) and Golgi Plug (catalog no. 555029, BD Biosciences).

### Cytokine Analysis

Supernatant cytokine analysis was performed on a Luminex 100/200 using the Millipore Milliplex MAP Murine Cytokine/Chemokine magnetic bead panel kit (catalog no. MCYTMAG-70k-PX32, Millipore/Sigma) and Curiox miniaturization protocol and hardware (LT-210MX-01-01, Curiox Biosystems).

### TPST-1495 Inhibition of Tumor Cell Growth *In Vitro*

Tumor cells were plated at 5,000 cells per well in a 96-well plate and incubated overnight to adhere. Cells were then exposed to increasing concentrations of TPST-1495 for 30 minutes before exposure to increasing concentrations of PGE2. Cells with drug + PGE2 were incubated for 24 or 48 hours before an 3-(4,5-dimethylthiazol-2-yl)-2,5-diphenyltetrazolium bromide (MTT) assay (catalog no. M6494, Invitrogen) was performed to assess relative cell viability and abundance.

### TPST-1495 Inhibition of Tumor Cell Growth *In Vitro* with Serum Starvation

Tumor cells were plated at 5,000 or 10,000 cells per well in a 96-well plate and incubated overnight in complete media to adhere. Complete media was aspirated, cells were washed once with PBS (CAT #14190-250, Gibco), before media with varying concentrations of FBS including 0%, 1%, 5%, and 10% FBS was added to the plate. Cells equilibrated in the FBS starved media conditions for 30 minutes at 37°C before adding increasing concentrations of TPST-1495 using an autodispenser (TECAN). Cells were incubated in drug and FBS-restricted media for 48 hours before a CyQuant XTT Cell Viability Assay (catalog no. X12223, Invitrogen) was performed following the manufacturer's protocol to measure cell viability and abundance.

### IHC

Histology of APC^min/+^ tumors was performed at CrownBio. Antibodies that were used for staining are listed in Table 1B. Histology of CT26 tumors was performed by Histowiz, LLC. Antibodies that were used for staining are listed in Table 1C.

**TABLE 1B:** Antibodies used for IHC of APC^min^^/+^ tumors

Antibody	Company	Catalog no.	Isotype	Type	Reactivity
CD4	Cell Signaling Technology	25229S	IgG	Rabbit IgG mAb	Mouse, Rat
CD8	Cell Signaling Technology	98941	IgG	Rabbit IgG mAb	Mouse
FoxP3	Cell Signaling Technology	12653S	IgG	Rabbit IgG mAb	Mouse

**TABLE 1C:** Antibodies used for IHC of CT26 tumors

Antigen	Clone	Catalog no.	Source
CD8α	4SM15	13-0808-82	Thermo Fisher Scientific
CD4	EPR19514	ab183685	Abcam
F4/80	BM8	14-4801-82	Thermo Fisher Scientific

### Statistical Analyses

Graphs and statistical analyses were made using GraphPad Prism 9.4.1 software (GraphPad Software). Student *t* test and ANOVA were utilized to perform analyses between two groups or more than two groups as necessary. Gibbs tests for outliers were used to exclude outlier data points as necessary. Statistical significance in Kaplan–Meier curves was determined with log-rank and Mantel–Cox analysis. All values from statistical analyses were reported as follows. ****, *P* < 0.0001; ***, *P* < 0.001; **, *P* < 0.01; *, *P* < 0.05.

### Data Availability

The sequencing data generated in this study are publicly available in Gene Expression Omnibus at series record GSE229806.

## Results

### Characterization of TPST-1495, a First-in-class Dual Antagonist of the EP2 and EP4

**FIGURE 1:**
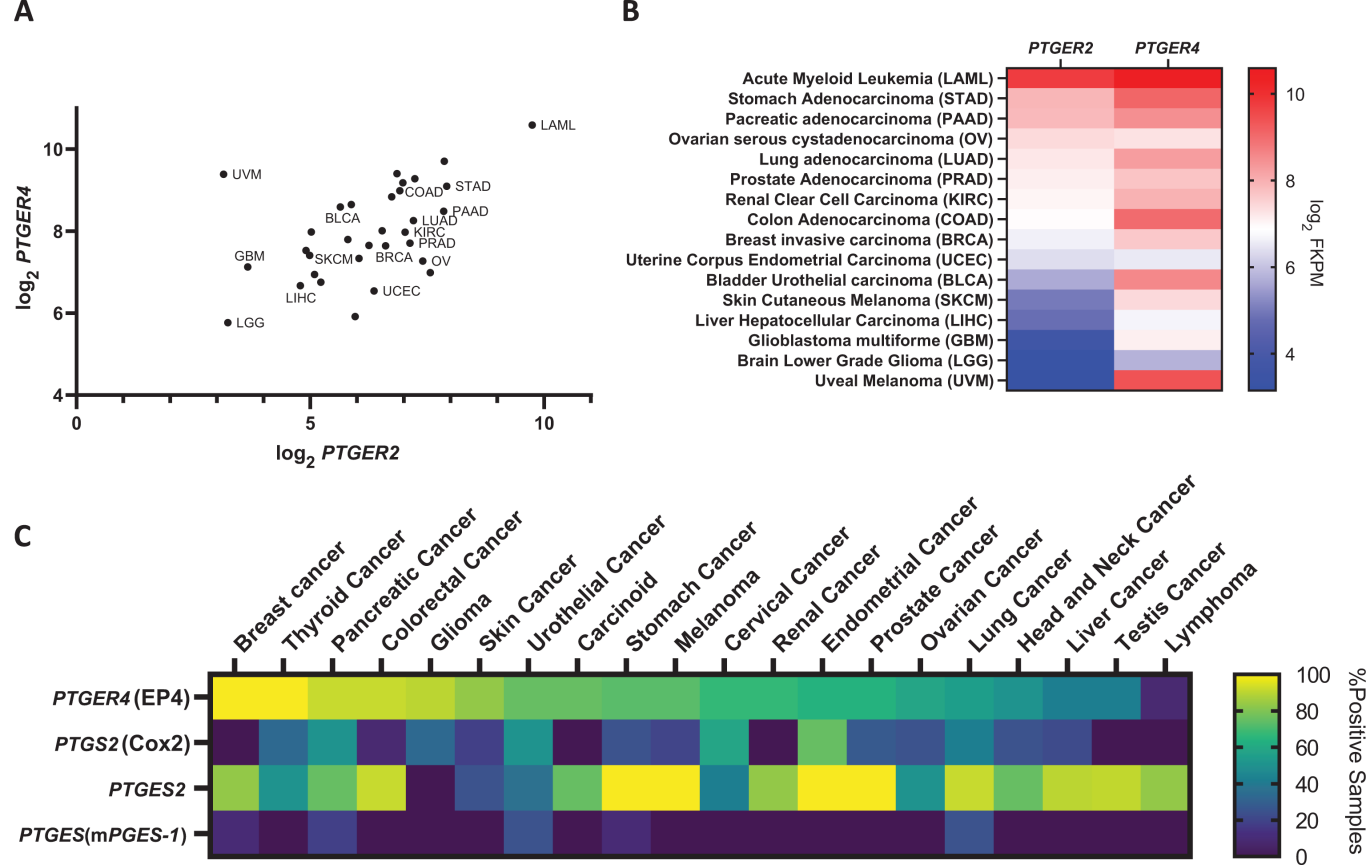
Expression profiles of PGE2 receptors EP2 and EP4 across diverse human malignancies. **A,** Expression of EP2 (*PTGER2*) and EP4 (*PTGER4*) across cancer indications indicated in the figure using RNA sequencing data from TCGA Pan Cancer dataset obtained through cBIO Portal. Selected cancer types are labeled with TCGA abbreviations. **B**, Heat map of EP2 and EP4 expression across the selected malignancies, with definition of TCGA tumor type abbreviations defined. **C**, Heat map analysis of percent positive samples for designated prostaglandin pathway genes, including *PTGER4*, *PTGS2*, *PTGES2*, and *PTGES* from the Protein Atlas database.

Analysis of The Cancer Genome Atlas (TCGA) using Cbio Portal revealed that EP2 was found to be expressed in diverse malignancies together with EP4 (Fig. 1A and B; ref. 33). Similarly, analysis of PGE2 pathway proteins from Protein Atlas demonstrated the abundance of several prostaglandin pathway enzymes and receptors (Fig. 1C; ref. 34). Given the immune-suppressive properties resulting from signaling through each of the EP2 and EP4 receptors, we endeavored to develop a selective dual antagonist. While selective EP2 antagonists (e.g., PF-04418948; Pfizer), and selective EP4 antagonists (e.g., E7046; Eisai Co., Ltd.) have been described in the literature, a balanced EP2/EP4 dual antagonist has not. In their investigation to develop a selective EP4 receptor antagonist (MK-2894; ref. 35), Blouin and colleagues described the EP4 single antagonist compound 26a which serendipitously also had some activity against the EP2 receptor but without any antagonist activity against the homologous EP1 and EP3 receptors. We first developed and characterized TPST-7131, a competitive antagonist with a 0.4 nmol/L IC_50_ value against EP4 receptor signaling, determined in a calcium flux assay against human EP4, using commercially available HEK293-based cell lines engineered to stably express individual designated EP receptors linked genetically with a promiscuous Gα subunit to couple each receptor to a calcium flux readout in response to PGE2 binding (36). Starting with the TPST-7131 lead, we then conducted a multiparameter lead optimization to generate the dual-selective EP2/EP4 receptor antagonist TPST-1495 with a key finding that presentation of the carboxylic acid containing moiety within the binding site can modulate the selectivity profile (37). TPST-1495 was evaluated as a competitive antagonist to PGE2 signaling in a calcium flux assay against human EP2 and EP4 PGE2 receptors in the engineered HEK293 cells described above. The calculated IC_50_ for EP2 was 17.21 and 3.24 nmol/L for EP4 (Supplementary Fig. S1). In the PGE2 competition assay conducted with HEK293-EP1 or HEK293-EP3 cell lines, TPST-1495 did not achieve 50% inhibition of EP1 or EP3 receptors at concentrations up to 30 μmol/L, and the IC_50_ values were calculated to be 134,200 nmol/L and 108,800 nmol/L for EP1 and EP3, respectively (Supplementary Fig. S1A). These results demonstrate that TPST-1495 is an exquisitely selective and specific dual antagonist of human EP2 and EP4 (Supplementary Fig. S1B). TPST 1495 demonstrated oral bioavailability and good exposure in animals, as did E7046 (Eisai LTD, co.), a single EP4 receptor antagonist in clinical development and used extensively in this investigation as a comparator (Supplementary Fig. S2). In addition, structural homology between murine and human EP receptors enabled the use of murine and human model systems to interrogate the activity of TPST-1495 herein.

### Dual Blockade of EP2 and EP4 PGE2 Receptors Overcomes PGE2-mediated Immune Suppression in Mouse and Human Monocytes *In Vitro*

**FIGURE 2:**
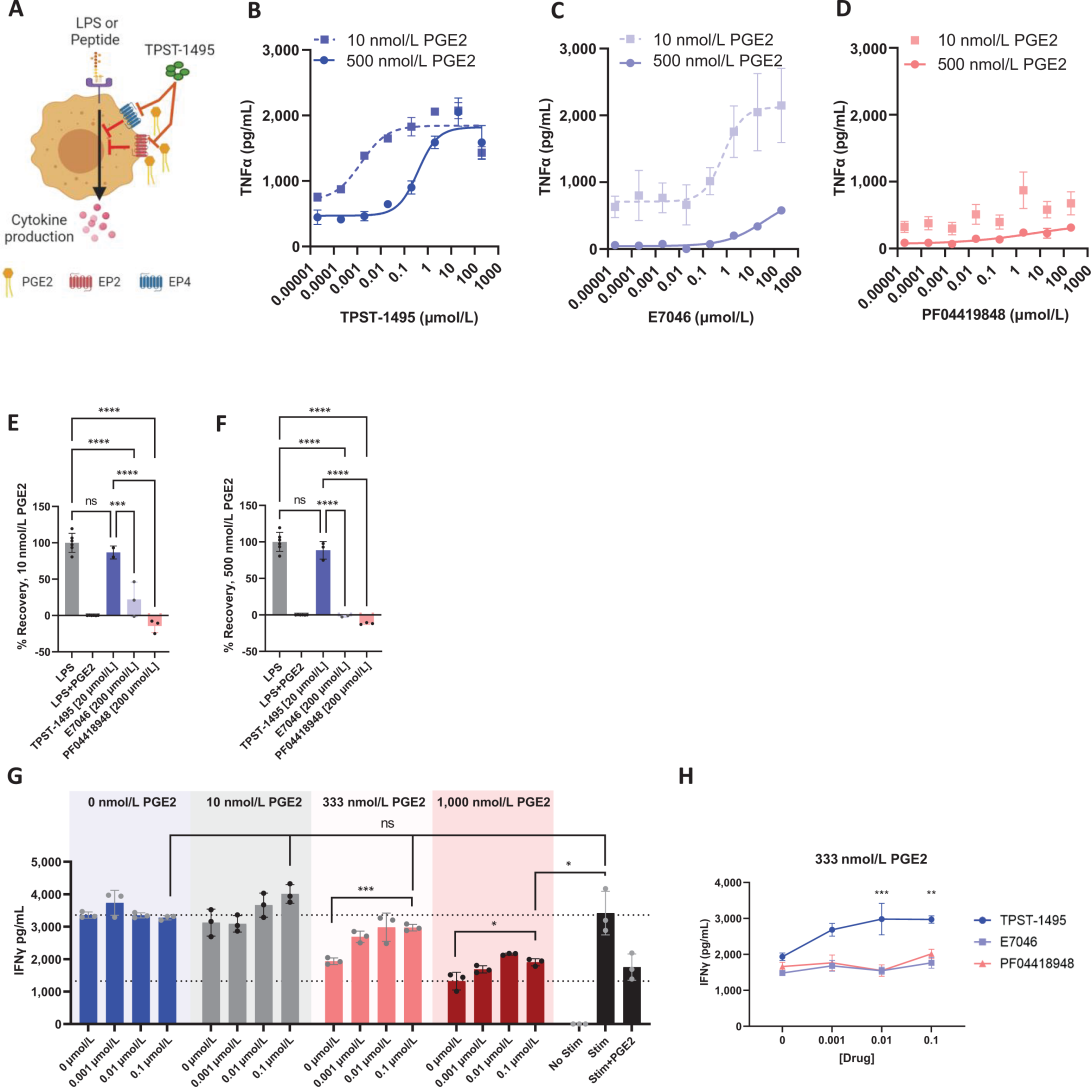
TPST-1495 is more effective than a single EP4 or EP2 antagonists in overcoming PGE2-mediated immune suppression of recovery of cytokine production from murine monocytes and T cells. **A,** Schematic of PGE2 blockade assay. Murine whole blood (**B**–**F**) or splenic and lymph node T cells (**G**, **H**) were collected and incubated with inhibitors for 30 minutes at 37°C, followed by incubation with PGE2 at labeled concentrations for 30 minutes at 37°C, followed by stimulation with LPS (0.5 μg/mL, overnight) or SIINFEKL peptide (2 μmol/L, 5 hours). Detection of TNFα production by ELISA (**B**–**D**) and maximum percent recovery (**E**, **F**) after blockade assay was performed on murine whole blood. Representative data shown from one of three experiments performed with *N* = 3 technical replicates per experiment. Some technical replicates failed to meet threshold TNFα values in low Stim groups. Percent recovery as calculated Percent recovery = 100*(“Experimental group” − “LPS+PGE2”)/(“LPS” − “LPS+PGE2”). **G** and **H,** IFNγ production detected by Luminex in culture supernatants after treatment with TPST-1495 across a PGE2 concentration gradient (**F**), or with 333 nmol/L PGE2 comparing multiple PGE2 receptor inhibitors (**G**) in the PGE2 blockade assay performed with a single-cell suspension of OT-1 splenic and lymph node isolates stimulated with SIINFEKL peptide. Data are representative of three experiments with *N* = 3 technical replicates per experiment. **H,** IFNγ production levels in OT-1 T cell cultures containing 333 nmol/L PGE2 and incubated with TPST-1495 EP2/EP4 dual antagonist, or EP2 (PF04418948) or EP4 (E7046) single antagonists. Mean and SD are depicted in all graphs and the ordinary one-way ANOVA with Tukey multiple comparisons test was used to assess significance between individual groups. ****, *P* < 0.0001; ***, *P* < 0.001; **, *P* < 0.01; *, *P* < 0.05.

To define the response of dual EP2 and EP4 antagonism in comparison to single antagonism of either receptor, we exploited the capacity of PGE2 to suppress downstream TNFα production from monocytes following stimulation with LPS (Fig. 2A; refs. 9, 38). We exposed mouse whole blood to a low concentration of PGE2 (10 nmol/L) that would effectively engage the EP4 but not the EP2 receptor, or with 500 nmol/L PGE2, a concentration that would ensure PGE2 signaling through both EP2 and EP4 receptors. The affinity of murine PGE2 for receptors EP2 and EP4 is 12 nmol/L and 1.9 nmol/L, respectively. TPST-1495 treatment rescued production of TNFα by treated monocytes at both low and high PGE2 concentrations with IC_50_ values of 1 nmol/L and 382 nmol/L, respectively (Fig. 2B). In contrast, E7046, a specific EP4 antagonist, rescued production of TNFα only in the presence of low PGE2 (Fig. 2C). PF04419848, a specific EP2 antagonist, was unable to induce TNFα at the PGE2 concentrations tested (Fig. 2D). TPST-1495 rescued >80% of TNFα production from PBMC at both 10 nmol/L PGE2 (Fig. 2E) and 500 nmol/L PGE2 (Fig. 2F), whereas E7046 and PF04419848 had less than 50% recovery at both concentrations of PGE2. We then profiled the activity of TPST-1495 in OT-1 CD8^+^ T cells to determine whether dual EP2/EP4 inhibition was required to overcome PGE2 suppression during CD8^+^ T-cell activation. During stimulation of lymphocyte cultures with SIINFEKL peptide, TPST-1495 rescued IFNγ production at PGE2 concentrations up to 333 nmol/L, and significantly increased IFNγ production at 1,000 nmol/L PGE2. (Fig. 2G). Single antagonism of either EP2 or EP4 alone in OT-1 T-cell cultures with 333 nmol/L PGE2 failed to recover IFNγ production (Fig. 2H).

**FIGURE 3:**
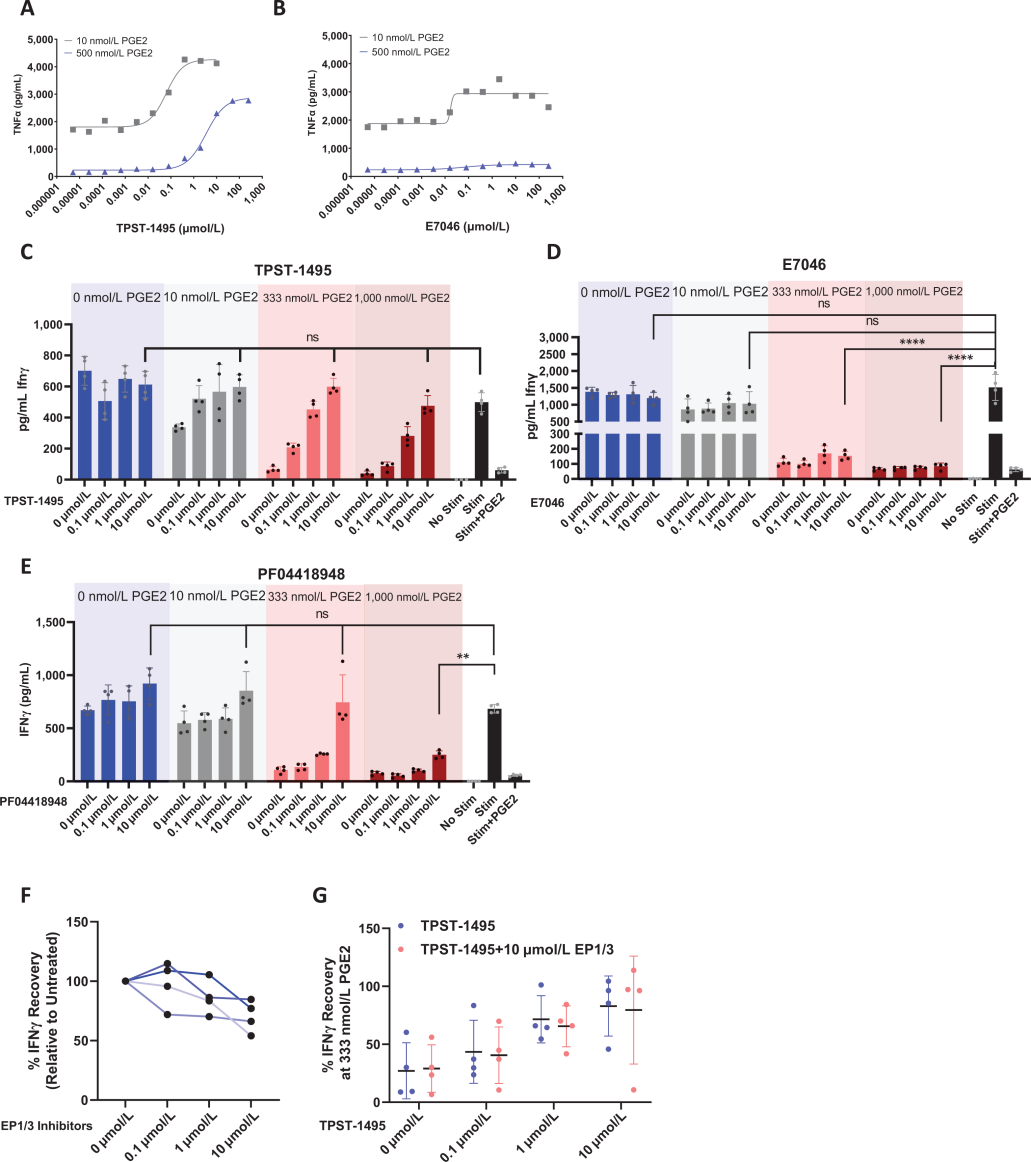
TPST-1495 is more effective than a single EP4 or EP2 antagonists in overcoming PGE2-mediated immune suppression of recovery of cytokine production from human monocytes and T cells. TNFα ELISA from PGE2 blockade assay performed using human whole blood treated with TPST-1495 (**A**) or E7046 (**B**) in the presence of 10 nmol/L or 500 nmol/L PGE2. Graphs show one representative donor of 3 donors tested. **C**–**E**, IFNγ concentration in supernatants of PGE2 blockade assay performed on magnetically enriched T cells from human PBMC. Cells were treated with various concentrations of EP receptor inhibitors TPST-1495 (**C**), E7046 (**D**), or PF04418948 (**E**), followed by concentrations of PGE2 and CEF peptides as shown in **C** and **D**. Cytokine levels in supernatants were determined by bead-based cytokine array analysis. Graphs show data with *N* = 4 technical replicates from one of four independent experiments. Two different healthy donors were used across these experiments. **F**, Percent IFNγ production rescue, as normalized to the control (DMSO), from PGE2 Blockade assay supernatants. Magnetically enriched T Cells from human PBMC were treated with increasing amounts of EP1 (Ono-8130) and EP3 (L-798106) inhibitors and subjected to 333 nmol/L PGE2 before stimulation with CEF peptides. Each datapoint represents the average percent recovery from one experiment, with four total experiments shown. **G**, Percent IFNγ production rescue, as normalized to control with 0 nmol/L PGE2 and 0 nmol/L TPST-1495. Cells obtained as in **C**–**F** were treated with increasing TPST-1495 in the presence or absence of 10 mmol/L EP1 inhibitor and 10 mmol/L EP3 inhibitor. Each datapoint represents the average percent recovery from one experiment, with four total experiments shown. Mean and SD are depicted in all graphs and the Student two-sided *t* test was used to assess significance between individual groups. ****, *P* < 0.0001; ***, *P* < 0.001; **, *P* < 0.01; *, *P* < 0.05.

We then tested whether our observations in mouse monocyte cultures would extend to human immune cells. PBMCs from healthy adult human donors were treated with TPST-1495 or E7046 antagonists in the presence of low (10 nmol/L) or high (500 nmol/L) PGE2. Consistent with our observations in mouse whole blood, treatment with TPST-1495 led to recovery of TNFα production at all concentrations of PGE2 tested (Fig. 3A). However, treatment with single EP4 blockade restored TNFα production only under low PGE2 conditions (Fig. 3B). We hypothesized that dual blockade of EP2 and EP4 receptors would be similarly required to overcome PGE2 suppression of human Ag-specific CD8^+^ T cells. Indeed, TPST-1495 treatment restored IFNγ production in PGE2 concentrations up to 1,000 nmol/L during stimulation with a peptide pool of common CD8^+^ T-cell epitopes from cytomegalovirus, Epstein–Barr virus, and influenza together (CEF; Fig. 3C). In contrast, the single EP4 antagonist E7046 did not rescue IFNγ production from CD8^+^ T cells in CEF-stimulated cultures at PGE2 levels above 10 nmol/L (Fig. 3D) and the single EP2 antagonist PF04419848 failed to rescue IFNγ production above 333 nmol/L PGE2 (Fig. 3E).

To determine whether EP1 and EP3 inhibition was detrimental to T-cell activation, we conducted the same T-cell activation assay adding combined EP1 and EP3 antagonists, either individually or in combination with TPST-1495. In the presence of 333 nmol/L PGE2, EP1, and EP3 single antagonists dampened IFNγ production in a dose-dependent manner (Fig. 3F). However, the addition of EP1 and EP3 antagonists to TPST-1495 did not consistently result in a decrease in IFNγ rescue as compared with TPST-1495 treatment alone (Fig. 3G). Collectively, our results demonstrate that dual inhibition of both EP2 and EP4 receptors in human PBMC cultures reverses PGE2-mediated immune suppression over a wide concentration range significantly more effectively than either single EP2 or EP4 receptor antagonists and that specific combined inhibition of EP2 and EP4 may be necessary to potentiate full CD8^+^ T-cell activity.

### TPST-1495 Confers Therapeutic Efficacy in Mice Bearing PGE2-Producing Tumors

**FIGURE 4:**
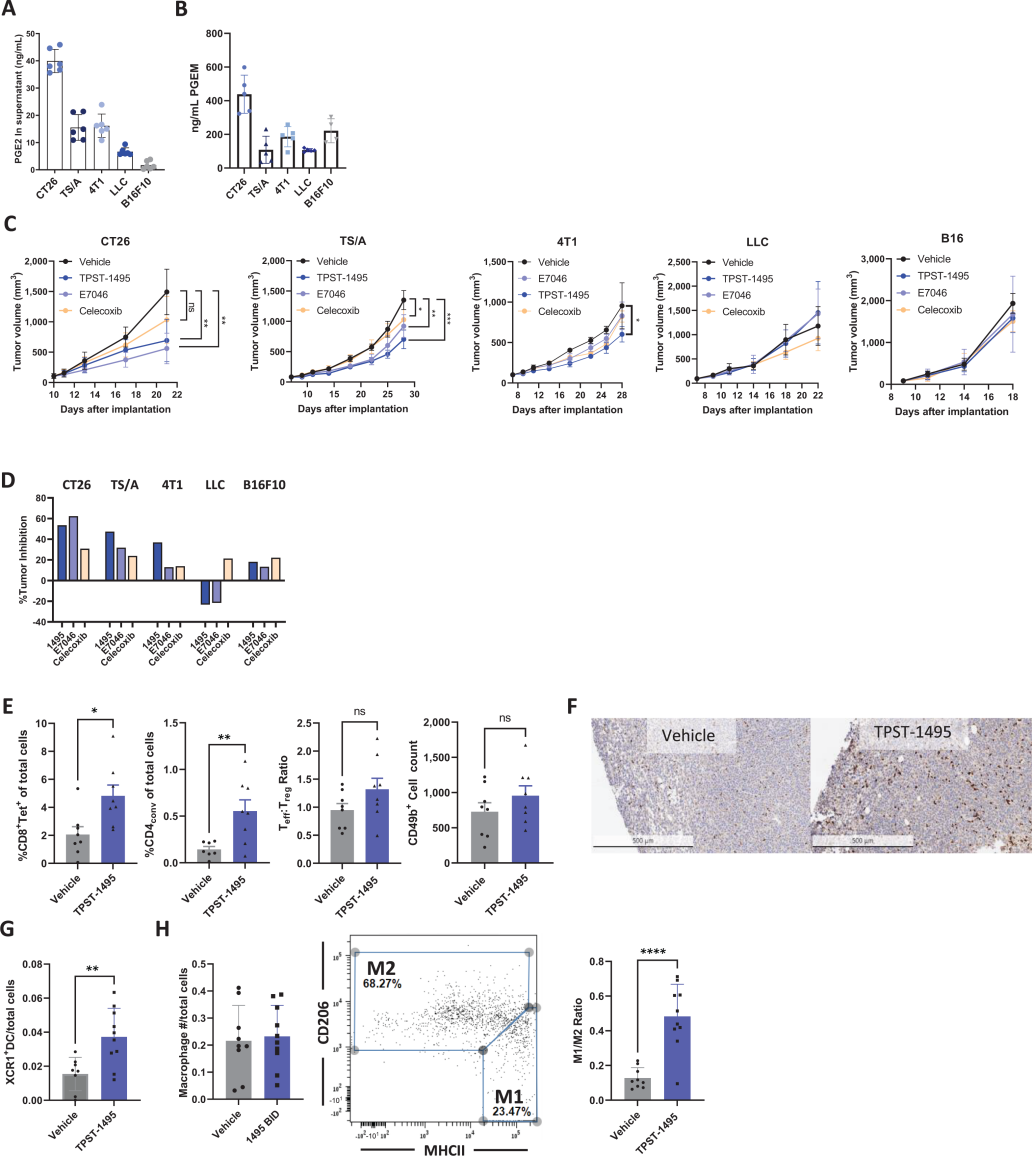
TPST-1495 confers therapeutic efficacy in mice bearing PGE2-producing tumors. **A,** PGE2 detected by mass spectrometry in cell culture fluids of tumor cells lines after 24 hours in culture. Results depict six technical replicates. **B,** PGEM detected by mass spectrometry in urine of tumor-bearing mice from untreated animals (shown in C) approximately 17 days after tumor implantation. Results are representative of one experiment with five biological replicates. Tumor outgrowth (**C**) and percent TGI [TGI = 100*(Control Volume − Treatment Volume)/Control Volume] (**D**) measured in mice bearing flank implanted tumors of tumor types shown in the figure. A total of 1 × 10^6^ cells were implanted on day 0 and treatment in mice randomized to assigned groups was initiated when average tumor volume within a tumor line reached approximately 80–100 mm^3^. Mice were treated with TPST-1495 (100 mg/kg twice a day, orally), E7046 (150 mg/kg every day, orally), PF04418948 (100 mg/kg every day, orally), or Celecoxib (60 mg/kg every day, orally) and followed for outgrowth until vehicle mice reached humane endpoints. Results are representative of two experiments with *N* = 5 mice per group. Mean and SD are depicted in all graphs and the Student two-sided *t* test was used to assess significance between individual groups at the last timepoint before mice were sacrificed. **, *P* < 0.01; *, *P* < 0.05. Flow cytometry (**E**) or IHC (**F**) from CT26 tumors after 14 days of treatment. A total of 1 × 10^6^ CT26 cells were implanted in the flanks of animals and followed until the average was 80–100 mm^3^. Mice were then treated for 14 days at which time tumors were resected and stained. Results depict one experiment with 8 mice, representative of more than three similar experiments. **G** and **H**, Flow cytometry from CT26 tumors resected after 17 days of treatment. Tumors were implanted as in **E**, treated for 17 days with TPST-1495, and then time tumors were resected and stained for DC1^+^ dendritic cells (G) or macrophages (H). Results shown in G represent one experiment with 10 mice, representative of multiple similar experiments with an average of 2-fold increase over vehicle, but data depicted are the only replicate that met *P* < 0.05 statistical cutoff. One datapoint in G met the Gibbs criteria for outliers and was excluded. Results in H represent one experiment of two similar experiments with at least 8 mice. Mean and SD are depicted in all graphs and the Student two-sided *t* test was used to assess significance between individual groups. ****, *P* < 0.0001; **, *P* < 0.01; *, *P* < 0.05.

Having shown the potency of dual blockade of EP2 and EP4 receptors in human and mouse immune cells *in vitro*, we then tested whether these findings translated to enhanced antitumor activity *in vivo*. Several tumor models were selected on the basis of a wide range of COX2 pathway activity, determined by measuring PGE2 levels in the supernatant of cultured tumor cells and PGEM levels in urine of tumor-bearing mice (Fig. 4A and B). TPST-1495 therapy at 100 mg/kg twice a day was initiated when tumors were 80–100 mm^3^, a dose level resulting in plasma exposures that exceeded the protein adjusted EC_50_ for rescue of TNFα production. TPST-1495 inhibited tumor growth in mice bearing colon carcinoma (CT26), triple-negative breast cancer (4T1), and mammary adenocarcinoma (TS/A), but not in those mice bearing LLC or B16F10 melanoma tumors (Fig. 4C). TPST-1495 exhibited a maximum tumor growth inhibition (TGI) of 54% in the CT26 model, 47% in the TS/A model, 37% in the 4T1 model, and 18% in the B16F10 model. In contrast, TGI in celecoxib-treated mice did not exceed 31% in any model tested (Fig. 4D). While TGI was generally higher among cohorts treated with TPST-1495 as compared with the single EP4 antagonist, E7046, these differences were not significant (Fig. 4D).

### Immune-Modulatory Effects of TPST-1495

Because combined inhibition of PGE2 signaling through EP2 and EP4 receptors significantly reversed PGE2-mediated immune suppression and due to previous work suggesting the importance of COX2 expression and immune inhibition (19), we interrogated components of the immune response to characterize mechanisms associated with TPST-1495 therapeutic benefit. BALB/c mice bearing established CT26 tumors were treated with TPST-1495 for 14 days at 100 mg/kg twice a day and the number of TILs were quantified by flow cytometry. TPST-1495 induced significant infiltration of tetramer-positive AH1-specific CD8^+^ T cells and CD4^+^ FoxP3^−^ cells into the tumor and moderately increased CD49b^+^ natural killer (NK)-cell infiltration and the ratio of tumor CD8^+^ to Treg^+^ T cells (Fig. 4E). Infiltration of CD8^+^ T cells in CT26 tumors was additionally measured by IHC staining (Fig. 4F), confirming values measured in Fig. 4E. In addition, we utilized the ovalbumin-expressing EL-4 tumor cell line EG-7 and confirmed that tumor-infiltrating T cells from animals treated with TPST-1495 were significantly more effective at producing cytokines after *ex vivo* stimulation with phorbol 12-myistate 13-acetate and ionomycin (Supplementary Fig. S3A–S3D). To further understand the role of T cells, tumor outgrowth was measured in CT26 tumor-bearing mice that were depleted of CD8α^+^ cells 2 days prior to initiating TPST-1495 therapy. In this context, after 14 days of TPST-1495 treatment therapy, tumor outgrowth was inhibited by 33% inhibition when T cells were depleted and 52% without T-cell depletion, suggesting that the observed anti-CT26 tumor response resulted from both T cell–dependent and T cell–independent mechanisms (Supplementary Fig. S4).

In addition to promoting increased lymphocytic infiltration into the TME, TPST-1495 treatment also resulted in an increase in the numbers of XCR1^+^ DC1 cross-presenting dendritic cells, as a proportion of total cells within the tumor (Fig. 4G). While macrophage cell numbers in the TME were largely unchanged in TPST-1495–treated mice, we found that macrophages were significantly shifted to an M1 effector phenotype, as indicated by an increase in MHCII^+^CD206^−^ macrophages as compared with vehicle-treated mice, which displayed a higher prevalence of the MHCII^low^ CD206^+^ M2 macrophage phenotype (Fig. 4H). Notably, TPST-1495 significantly enhanced PD-1 therapeutic efficacy in mice bearing CT26 flank tumors, suggesting that primed T-cell responses were potentiated by the combination of PD-1 and PGE2 blockade (Supplementary Fig. S5). Collectively, these results demonstrate that treatment of mice bearing high PGE2-producing CT26 tumors with TPST-1495 remodeled the TME as shown by a significant increase of innate and adaptive effector immune cell populations, combined with the reduction of immune suppressive innate and adaptive immune cell populations and significant therapeutic benefit when combined with immune checkpoint inhibitors.

### TPST-1495 Exhibits Immune-Independent Therapeutic Antitumor Activity

**FIGURE 5:**
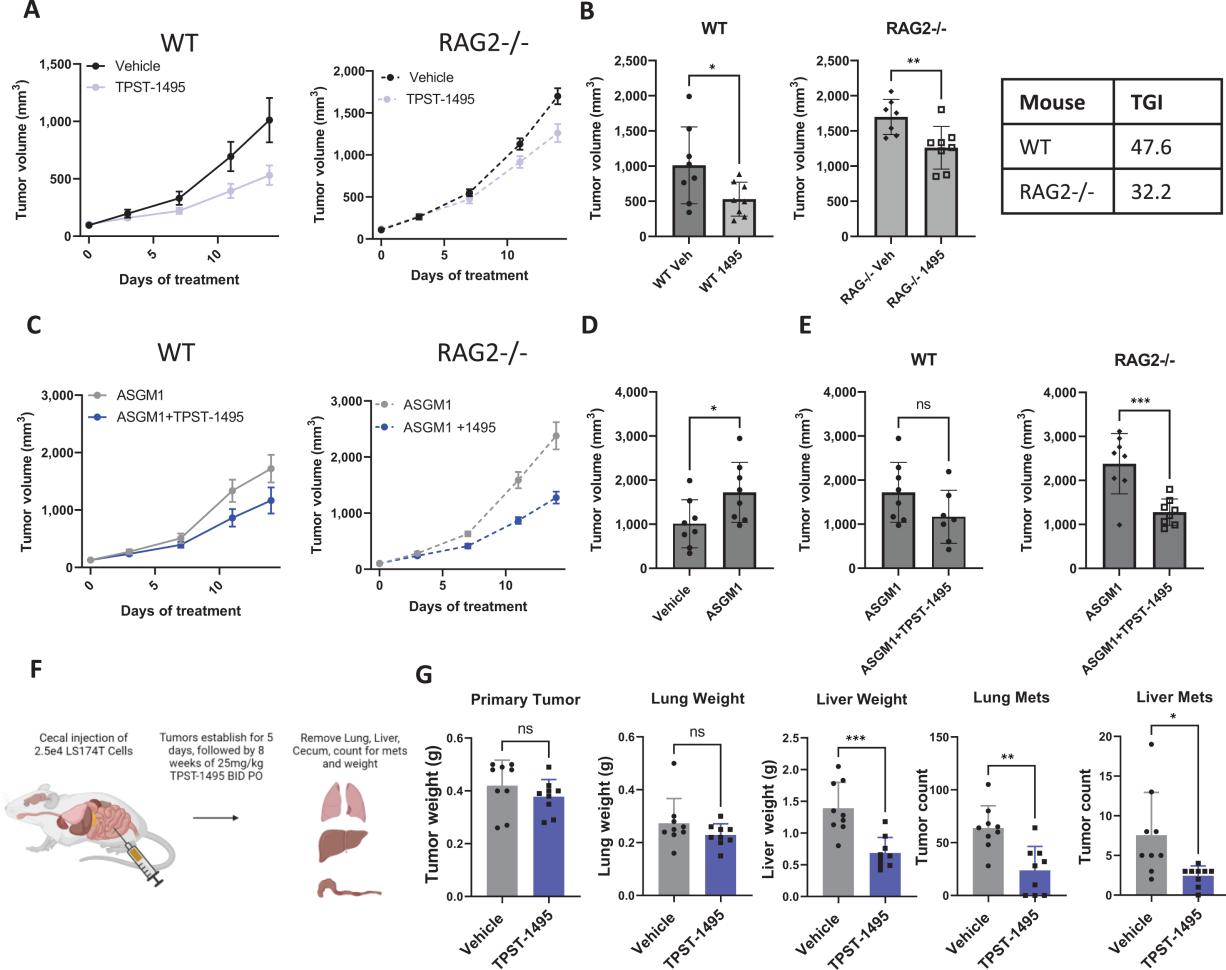
TPST-1495 exhibits immune-independent antitumor activity. CT26 tumor longitudinal outgrowth of WT and RAG2^−/−^ mice treated with 100 mg/kg TPST-1495 BID for 2 weeks (**A**) and day 14 volumetric comparisons between mouse groups (**B**). Results are representative of three experiments with at least 8 mice per experiment. CT26 tumor longitudinal outgrowth (**C**) and day 14 volumetric comparisons (**D** and **E**) in WT and RAG2^−/−^ mice treated with 100 mg/kg TPST-1495 twice a day for 2 weeks and 10 μL anti-asialo-GM1 antibody. **F**, Experimental schematic of LS174T colorectal xenograft model and tumor growth results. **G**, Enumeration of tumor burden by primary tumor weight, metastatic organ weight, or count of metastatic nodules. Results are representative of two experiments with at least 8 mice per experiment. Mean and SD are depicted in all graphs and the Student two-sided *t* test was used to assess significance between individual groups. ns, not significant; ***, *P* < 0.001; **, *P* < 0.01; *, *P* < 0.05.

In addition to its immunosuppressive capacity, other tumor-extrinsic properties of PGE2 include its support of the TME by promoting angiogenesis as well as tumor-intrinsic properties of driving tumor cell survival (11, 39–41). The diverse tumor-promoting properties of PGE2 prompted us to test whether inhibition of immune-independent mechanisms might also play a role in the observed antitumor activity of TPST-1495. To assess this possibility, we evaluated the antitumor activity of TPST-1495 in RAG2^−/−^ and NSG genetically defined immune-deficient mice. TPST-1495 treatment inhibited the outgrowth of CT26 flank tumors in both RAG2^−/−^ and wild-type (WT) mice, though to a lesser degree in the absence of adaptive T-cell immunity (Fig. 5A and B). As it has been shown previously that tumor-infiltrating NK cells play a critical role in initiating antitumor immunity in response to COX-2 pathway inhibition, we tested whether NK depletion would reduce the therapeutic effect of TPST-1495. We found that while NK-cell depletion increased tumor outgrowth, TPST-1495 therapy was similarly efficacious in WT and RAG^−/−^ tumor-bearing mice that were depleted of NK cells, demonstrating that the mechanism of TPST-1495 antitumor activity was both NK cell and T cell independent (Fig. 5C–E). To further test this possibility, we utilized LS174T human colon tumor cells, which harbor a PIK3CA mutation that enhances COX-2 expression and signal transduction and tumor proliferation through autocrine signaling of PGE2 through EP2 and EP4 receptors. LS174T tumor cells were orthotopically implanted into the cecum of NSG mice (Fig. 5F), which, due to IL2rg deficiency along with NOD and SCID mutations, lack T, B, and NK cells in addition to hyporesponsive innate immune cells. TPST-1495 treatment did not significantly inhibit primary tumor growth in the cecum, but tumor metastasis to both the lung and liver was significantly inhibited, as indicated by both tumor number and tissue weight as compared with the nontreated mice (Fig. 5G). We have not observed significant antiproliferative properties of TPST-1495 when added to cultured tumor cells, tested at drug levels sustained in the periphery of animal experiments (Supplementary Fig. S6).

### TPST-S1495 Effectively Increases Survival and Modulates the TME in APC^min/+^ Mice

**FIGURE 6:**
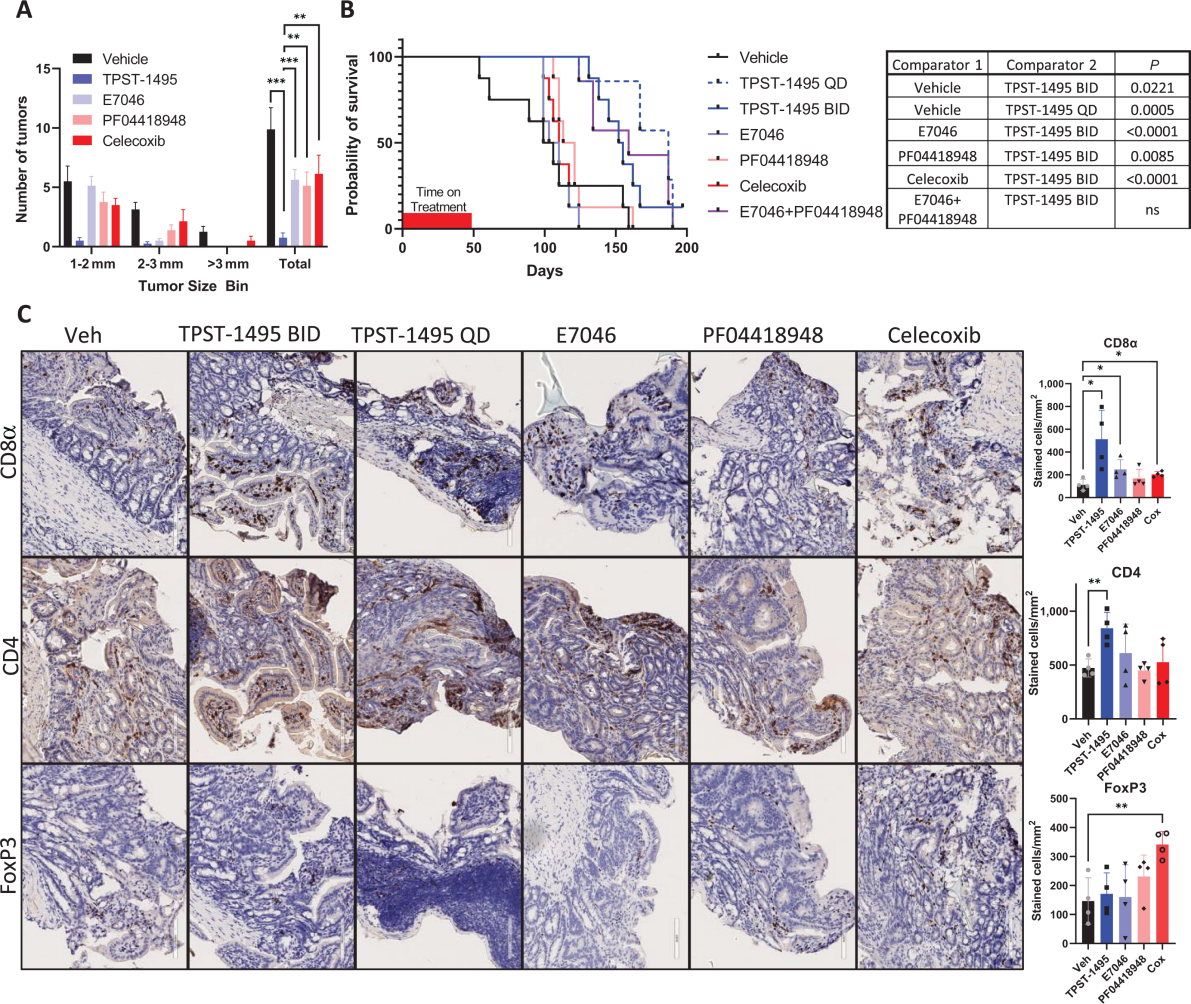
TPST-1495 effectively increases survival and modulates the TME in APC^min/+^ mice. **A,** Tumor counts in the small intestine of APC^min/+^ mice. Mice were treated starting at 13 weeks of age for 3 weeks with TPST-1495 (100 mg/kg twice a day, orally), E7046 (150 mg/kg every day, orally), PF04418948 (100 mg/kg every day, orally), or Celecoxib (60 mg/kg every day, orally). Results are representative of multiple experiments with 7–10 mice. **B**, Kaplan–Meier curve of survival of APC^min/+^ mice and log-rank Mantel–Cox *P* values of listed comparisons. Mice were ages to 13 weeks, then treated with as in A or with the combination of E7046 and PF04418948 at monotherapy doses, for 6 weeks. Survival was monitored longitudinally and mice were sacrificed as they became moribund. Results are representative of two similar experiments with 8 mice per group. **C**, IHC of tumor or hyperplasia excised from small intestines of mice from experiment in A. Quantification displays values from one tumor in each biological replicate. TPST-1495 every day and twice a day groups are concatenated because of limited tumor tissue in these groups. Mean and SD are depicted in all graphs and the Student two-sided *t* test was used to assess significance between individual groups. ****, *P* < 0.0001; ***, *P* < 0.001; **, *P* < 0.01; *, *P* < 0.05. BID, twice a day; QD, every day.

APC^min/+^ mice are a genetically engineered mouse model (GEMM) that harbor a heterozygous mutation in the adenomatous polyposis coli gene and spontaneously develop colon adenomas resulting from activating signal transduction through the β-catenin pathway and e-cadherin, recapitulating human disease. Previous research demonstrated that PGE2 administration accelerates disease progression in APC^min/+^mice by promoting tumor cell growth, vascular formation, and immune suppression (39, 42–44). We tested the antitumor efficacy of TPST-1495 in 12 to 13 weeks old APC^min/+^ mice compared with other prostaglandin pathway inhibitors. Untreated mice at this age contained multiple tumors largely confined to the small intestine that measured >1 mm in diameter. After a 3-week course of therapy, we observed that mice given TPST-1495 had significantly lower adenoma burden in the small intestine as compared with mice treated with other PGE2 pathway inhibitors tested or control mice (Fig. 6A). We then sought to expand the therapeutic regimen in the APC^min/+^ mouse model and compare the longitudinal effects of an extended treatment regimen among the various prostaglandin pathway inhibitors. APC^min/+^ mice were given a 6-week course of therapy and monitored for survival. The extended treatment regimen significantly increased the survival of mice treated with TPST-1495 given every day or a twice a day dosing regimen. In contrast, treatment of APC^min/+^ mice with celecoxib or with single EP2 or EP4 PGE2 receptor antagonists did not significantly increase survival (Fig. 6B). To ensure that our dosing strategy for EP2 and EP4 single antagonists were effective at blocking their respective receptors, we combined single EP2 and EP4 receptor antagonists and observed that their *in vivo* activity was similar to that of TPST-1495. To further these observations, we tested the same treatment regimens in ovalbumin-expressing EG7 tumor cells and observed similar patterns of tumor regression and immune activity (Supplementary Fig. S3A–S3D) between TPST-1495 and combined single EP2/EP4 receptor antagonists. Consistently, TPST-1495 and combined single EP2/EP4 receptor antagonists showed similar potency in overcoming PGE-2-mediated immune suppression in human enriched T cells, though not whole blood monocytes (Supplementary Fig. S3E and S3F).

To determine whether our observations of differing efficacy in APC^min/+^ mice among the prostaglandin pathway inhibitors we tested aligned with differences in TME immune infiltrates, we measured lymphocyte populations in APC^min/+^ mice by performing IHC on resected small intestinal areas with tumors or hypertrophy after 3 weeks of prostaglandin pathway inhibitor treatment. Strikingly, at this timepoint, no adenomatous or carcinomatous tissue could be detected in TPST-1495–treated animals by blinded independent pathology assessment (Supplementary Fig. S7). In contrast, either no or a minimal decrease in the number of adenomas and carcinomas was observed in the small intestine of APC^min/+^ mice treated with COX-2 or single EP2 or EP4 PGE2 receptor inhibitors. Following the 3-week course of therapy, IHC staining revealed a significant increase in tissue-infiltrating CD8^+^ T cells in hypertrophic areas of the small intestine in mice treated with TPST-1495, E7046, and celecoxib, though not PF04418948 (Fig. 6C). While the level of CD8^+^ T-cell infiltration in TPST-1495–treated mice was greater than with other agents, these differences were nonsignificant. However, CD4^+^ T-cell infiltration was significantly higher in TPST-1495–treated mice. Interestingly, we observed that FoxP3 staining of CD4^+^ T cells increased uniquely in celecoxib-treated mice, suggesting that the increase of CD4^+^ T cells in TPST-1495–treated animals did not include an abundance of regulatory T cells.

### Effective Antagonism of PGE2 Signaling Modulates Significant Transcriptional Shifts in the TME

**FIGURE 7:**
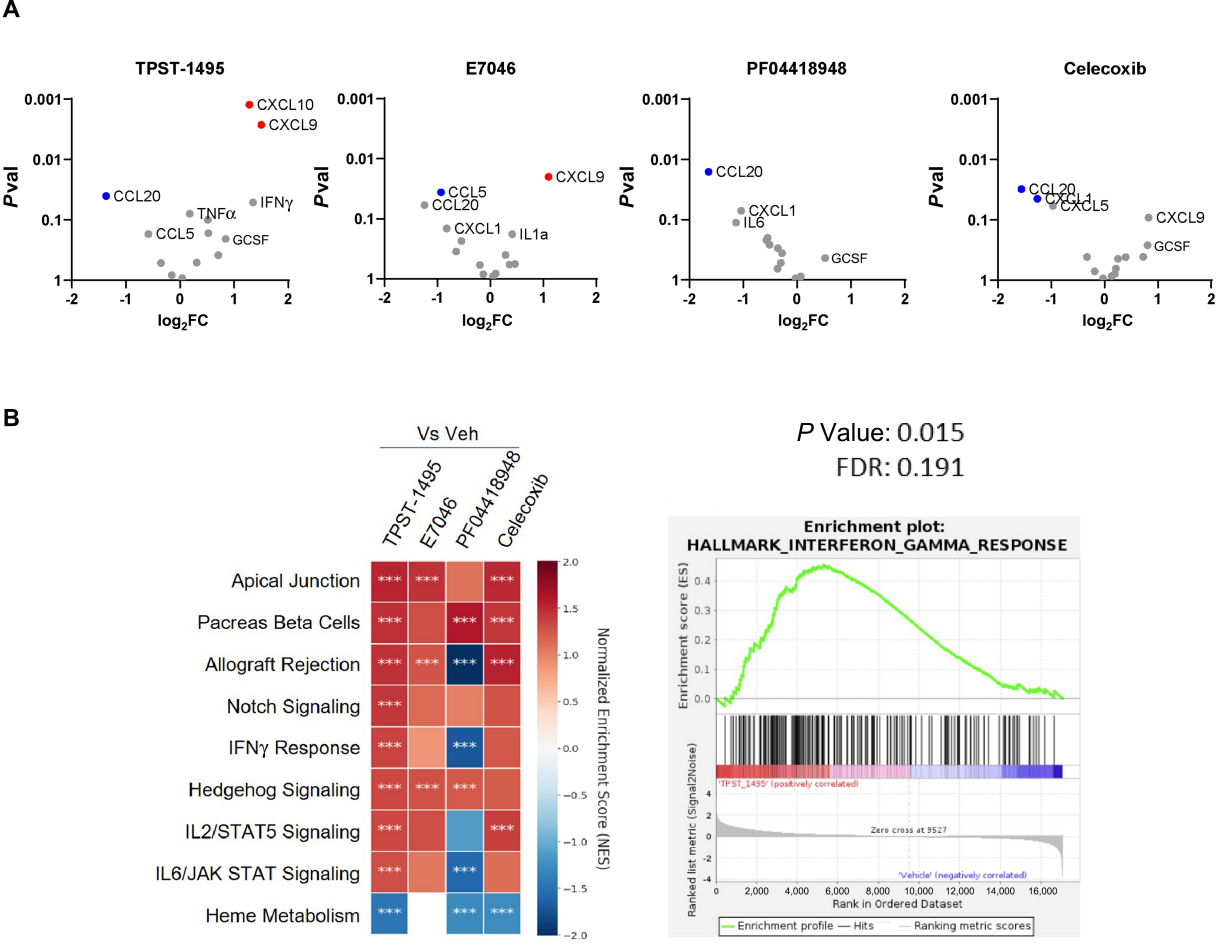
Effective antagonism of PGE2 signaling modulates significant transcriptional shifts in the TME. **A,** Volcano plots of selected genes. Blue coloration represents significantly (*P*_adj_ < 0.05) downregulated genes while red correlation represents significantly upregulated genes. Genes in gray did not meet the significance cutoff. **B**, Heat map depicting significantly upregulated gene sets from GSEA performed on RNA sequencing analysis above and representative GSEA plot from IFNγ signature. All results represent data from one experiment with 4 animals per group. *** indicates FDR < 0.25 for that gene set compared with vehicle-treated animals.

We show here that redundancy between EP2 and EP4 signaling makes single EP receptor blockade necessary but not sufficient to prevent PGE2 mediated immune suppression over a broad concentration range of prostaglandin. To compare the transcriptional profile of tumors treated with TPST-1495 to those treated with single EP antagonists or COX2 inhibitors, we performed RNA sequencing on hyperplasias resected from 16-week-old APC^min/+^ mice that were given a 3-week treatment course of TPST-1495, E7046, PF04419848, or celecoxib. We then curated a gene set by concatenating genes found to be significantly perturbed in publications describing transcriptional changes after genetic or pharmacologic inhibition of the PGE2 pathway [Fig. 7A (19, 21, 26)]. We found that TPST-1495 treatment upregulated or downregulated several genes from this curated set that was similar to published literature, most notably CXCL9, CXCL10, IFNγ, CCL20, and CCL5. However, E7046, PF04419848, and celecoxib treatment led to fewer and lower magnitude perturbations in this gene set, along with additional genes (Supplementary Fig. S8), demonstrating that EP2 and EP4 combined antagonism more effectively blocked inhibitory effects of PGE2. This conclusion was further supported by gene set enrichment analysis (GSEA), in which the IFNγ response signature was only significantly enriched in TPST-1495–treated samples (Fig. 7B).

## Discussion

In this investigation, we characterized the activity and mechanism of TPST-1495, a first-in-class dual antagonist which specifically inhibits signaling through the tumor-promoting EP2 and EP4 PGE2 receptors and allows for continued of PGE2 signaling through the immune-promoting EP1 and EP3 receptors. We show that this specific dual antagonism results in reversal of PGE2-mediated immune suppression and significantly increased immune activation of both mouse and human immune cells in vitro over broad concentrations of PGE2 as compared with COX2 or single EP2 or EP4 receptor inhibitors.

To evaluate the capacity of targeted and selective EP receptor blockade to overcome PGE2-mediated immune suppression, we developed innate and adaptive immune cell systems to measure recovery of immune activation using effector cytokine readouts. Production of TNFα by monocytes in response to LPS stimulation could be completely suppressed with PGE2, which increases intracellular cAMP levels and in turn inhibits NFκB activation and TNFα production. Addition of TPST-1495 recovered production of TNFα in human and mouse monocytes stimulated with LPS at both low (10 nmol/L) or high (500 nmol/L) PGE2 concentrations. Strikingly, recovery of TNFα production with a single EP4 receptor antagonist was observed with low levels of 10 nmol/L PGE2, but not at the high 500 nmol/L PGE2 concentration tested. PGE2 has a relatively low affinity for EP2 (*K*_d_ = 12 nmol/L in mouse, 13 nmol/L in humans) compared with EP4 (*K*_d_ = 1.9 nmol/L in mouse, 0.6 nmol/L in humans). Thus, the EP2 receptor was likely not effectively engaged in culture conditions with low PGE2 concentrations. In contrast, the EP2 receptor was engaged in high PGE2 concentration conditions, meaning that reversal of PGE2-induced immune suppression could be effectively reversed only with the dual EP2 and EP4 antagonist, TPST-1495 (Figs. 2 and 3). Interestingly, while the downstream signaling from EP2 and EP4 receptors is in part redundant, the differentiated IC_50_ values of these receptors suggests the possibility for rheostat control in which the extent of signaling is responsive to PGE2 concentration, perhaps important in the TME where there can be a broad range of PGE2 concentration. To demonstrate that blockade of both EP2 and EP4 receptor signaling was also essential for recovery of T-cell function in the presence of PGE2, we stimulated human and murine T cells with cognate peptide antigens for CEF responses in human cells or chicken egg ovalbumin (SIINFEKL, murine OT-1 cells) and measured both TNFα and IFNγ cytokine production as a function of various EP antagonists and PGE2 concentrations. In agreement with our monocyte assay results, blockade of both EP2 and EP4 by TPST-1495 was required for full recovery of CD8^+^ T-cell effector function at high PGE2 levels (Figs. 2 and 3). Significantly, our observation that adding EP1 and EP3 receptor antagonists inhibited activation of CD8^+^ T cells provides a central scientific rationale for selectively targeting both EP2 and EP4 receptors (while retaining PGE2 signaling through EP1 and EP3 receptors) as a more effective approach to overcome PGE2 immune suppression than blockade of PGE2 production with COX-2 inhibitors (e.g., celecoxib). Thus, in addition to increased immunologic potency, TPST-1495 conceptually provides a safety advantage over NSAIDs, which through blocking all PGE2 production result in thromboxane and prostacyclin imbalances leading to dose-limiting cardiac and renal toxicities.

While there are several investigations exploring the activity of prostaglandin targeted therapies in immunogenic and immune checkpoint blockade responsive tumor cells, there are fewer examples testing the activity of this agent class in tumor settings that are not T cell–inflamed and not responsive to immune checkpoint therapy. We chose to study the possible differentiated therapeutic activity of TPST-1495 in the spontaneous APC^min/+^ model of polyposis and colorectal cancer for several reasons, which we posit underline the importance of dual EP2 and EP4 receptor blockade: (i) prostaglandin signaling is known to be a significant driver of colorectal cancer; (ii) the APC^min/+^ model is spontaneous and less inflammatory than conventional implanted syngeneic tumor cells; and (iii) early clinical development involves studies in advanced patients that are refractory to or become refractory to immune checkpoint inhibition. We observed striking and significantly better potency of TPST-1495 compared with all prostaglandin pathway inhibitors tested in tumor-bearing APC^min/+^ mice. At 13 weeks, APC^min/+^ mice have established large and small intestine adenomas. TPST-1495 treatment for 3 weeks in 13-week-old APC^min/+^ mice led to near complete regression of gut disease determined by gross dissection and by blinded histopathology analysis (Fig. 6; Supplementary Fig. S7). In comparison, single EP receptor antagonists or celecoxib led only to partial tumor reduction. These results differed somewhat from those using subcutaneously implanted tumor models (Fig. 4C), in which EP4 inhibition by E7046 was not statistically distinguishable from dual EP2/4 inhibition by TPST-1495. APC^min/+^ and subcutaneous models used here differ significantly by multiple parameters including etiology, immune infiltration, and time on treatment. Further studies will be required to define treatment differences in these models.

Transcriptome analysis of APC^min/+^ tumors reinforced our understanding of the mechanism of action (MOA) (as defined by *in vitro* stimulation and *in vivo* depletion and GEMM studies) differences between TPST-1495 and other PGE2 blockade approaches. While transcriptional changes induced by TPST-1495 treatment were similar to those induced by single EP4 blockade, the changes were higher in magnitude. This observation suggests that redundant signaling of PGE2 through the EP2 receptor reduces the effect of single EP4 receptor blockade, for example, EP4 blockade is necessary but insufficient, and supports our proposed rheostat control model. The EP2 single antagonist had very little effect on immune cell activity, tumor regression, and the tumor transcriptome, reflecting the relatively low expression and affinity for PGE2 of this receptor compared with EP4.

Unique to our study, we demonstrated that TPST-1495 significantly inhibited metastasis of PIK3CA-mutant human LS147T tumors in immune-deficient NSG mice (Fig. 5). We chose this model due to observations that the PIK3CA mutation enhances COX-2 expression and promotes tumor proliferation via autocrine signaling, possibly informing eventual selection of patient populations that may be responsive to TPST-1495 therapy (22). While we did not define the mechanism underlying this result, we hypothesize the TPST-1495 either directly inhibited tumor proliferation required for metastasis and/or inhibited CXCL1-dependent vascularization of tumors, a known tumor-extrinsic tumor pathway promoted by PGE2 (39). LS174T cells undergo reduced apoptosis in serum-free conditions when exposed to increasing amounts of PGE2, but to our knowledge there are no analogous studies performed using *ex vivo* monoculture of isolated APC^min/+^ neoplasms to test the sensitivity of these adenomas and carcinomas to PGE2. Murine syngeneic transplantable models we tested did not reveal changes in proliferation or cytotoxicity of TPST-1495 treatment over a range of serum conditions (Supplementary Fig. S6). However, we have not ruled out the possibility that the requirement of PGE2 for tumor cell growth and survival is not well modeled *in vitro*, and that other cellular and environmental pressures necessitate PGE2 signaling *in vivo*, but not *in vitro*. Collectively, the experimental results support our assertion that the antitumor activity of TPST-1495 is due both to inhibition of tumor-intrinsic PGE2 signaling and inhibition of tumor-extrinsic PGE2-mediated suppression of the immune response.

TPST-1495 represents a unique approach to inhibit PGE2-driven malignancies and possibly increase the efficacy of immune checkpoint inhibitors. Supported by the experimental results presented here, we are evaluating the safety, tolerability, pharmacokinetics/pharmacodynamics, and possible antitumor activity in a dose escalation, optimization and scheduling clinical phase Ia/Ib study, both as monotherapy and in combination with pembrolizumab, in patients with advanced solid tumors who have failed available standard-of-care therapies (NCT04344795). The results shown in this investigation also support the rationale for testing TPST-1495 in familial adenomatous polyposis coli, in which individuals carrying the APC^min/+^ germline mutation have nearly a 100% lifetime risk for developing colorectal cancer.

## Supplementary Material

Supplementary Figures S1-S8 Supplementary Figures S1-S8
