# Supplementary material for: Dual Blockade of EP2 and EP4 Signaling is Required for Optimal Immune Activation and Antitumor Activity Against Prostaglandin-Expressing Tumors
Source: Cancer Res Commun. 2023 Aug 8;3(8):1486–500. doi: 10.1158/2767-9764.CRC-23-0249 (PMC10408683; doi:10.1158/2767-9764.CRC-23-0249)
Supplement: Supplementary Figures S1-S8 [file crc-23-0249-s01.pdf]

Figure S1: EP receptor antagonist activity and specificity

A

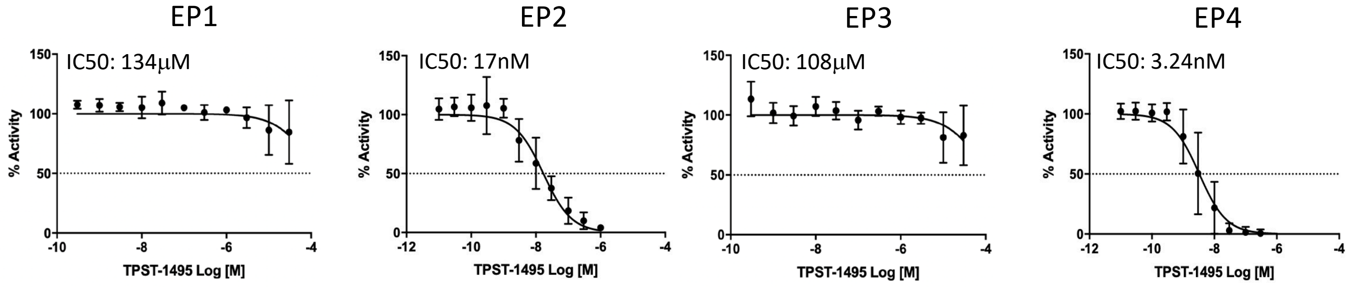

B

	Prostaglandin E2 receptor			
	EP1	***EP2***	***EP3***	***EP4***
**Replicates**	n = 2	n = 14	n = 2	n = 15
**IC_{50} nM**	134,200	17.21	108,800	3.24
**95% CI**	73,420 to 385,000	14.81 to 20.04	60,840 to 275,400	2.86 to 3.66

Figure S1: TPST-1495 EP receptor antagonist activity and specificity

A) **TPST-1495 EP receptor PGE2 antagonist values in CA++ flux assays.** Serial eleven-point half-log dose response curves shown in 293HEK cells engineered to express a single designated EP receptor linked genetically to a promiscuous G alpha coupled to calcium flux (Eurofins Scientific). Prostaglandin PGE2 was used at a final concentration of 10 nM for EP1, EP2 and EP4 assay, and 200 nM for EP3 assay. Error bars represent standard deviation.

B) **TPST-1495 EP receptor IC50 antagonist values.** Percent activity of TPST-1495 was determined against the average of the positive control (100%) and negative control (0%). The IC_{50} of TPST-1495 for EP receptors was determined as log inhibitor against normalized response (variable slope) curve fitting. The number of experiments performed to determine the antagonist activity against each EP receptor is shown (as n = x) in the Table. IC_{50} was determined for each individual experiment. Composite IC_{50} was determined using the normalized percent activity per experiment results.

Figure S2: Pharmacokinetic (PK) and exposure characterization of TPST-1495 and E7046

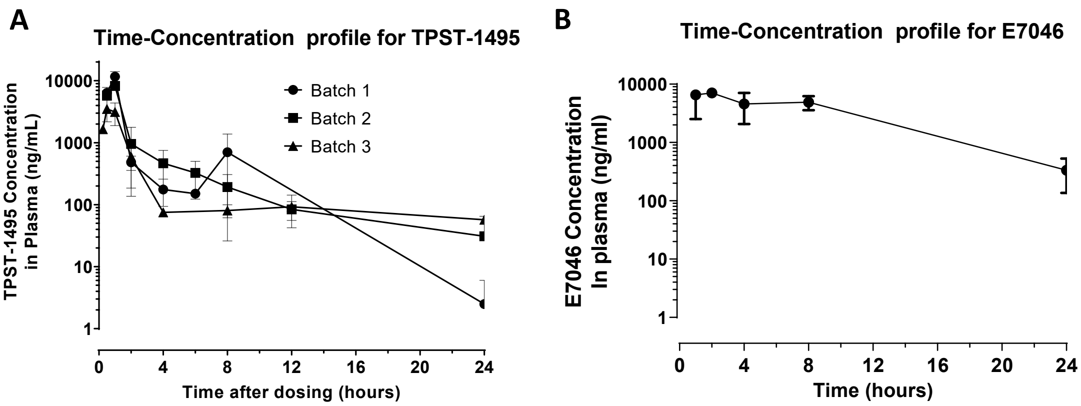

C

Parameter						
Route of Adminstration	PO	PO	PO	PO	PO	PO
Test Article	1495	1495	1495	1495	1495	E7046
Mouse strain	Balb/c	Balb/c	Balb/c	C57Bl/6	C57Bl/6	Balb/c
Number of mice per timepoint	2	2	2	2	5	2
Dose (mg/kg)	100	100	100	100	100	30
Batch #	5A	13A	15A	15A	15A	
AUC_{0-t} (µg*h/mL)	19.6	13.5	6.58	7.96	5.72	82.5
T_{max} (h)	1.0	1.0	0.5	1.0	0.5	1.5
C_{max} (µg/mL)	11.6	8.22	3.50	5.12	2.74	8.0
C_{max} (µM)	23.2	16.4	6.99	10.2	5.47	16.5

Figure S2: Pharmacokinetic (PK) and exposure characterization of TPST-1495 and E7046

A) Measured TPST-1495 plasma concentration after treatment with 100mg/kg PO of 3 independently produced batches of TPST-1495. Each line represents one batch and contained two mice. Error bars represent standard deviation.

B) Measured E7046 plasma concentration after treatment with 30mg/kg PO administration of a single batch of commercially obtained material. The data depicts the average of two mice and error bars represent standard deviation.

C) Data table of individual PK parameters calculated from (A,B).

Figure S3

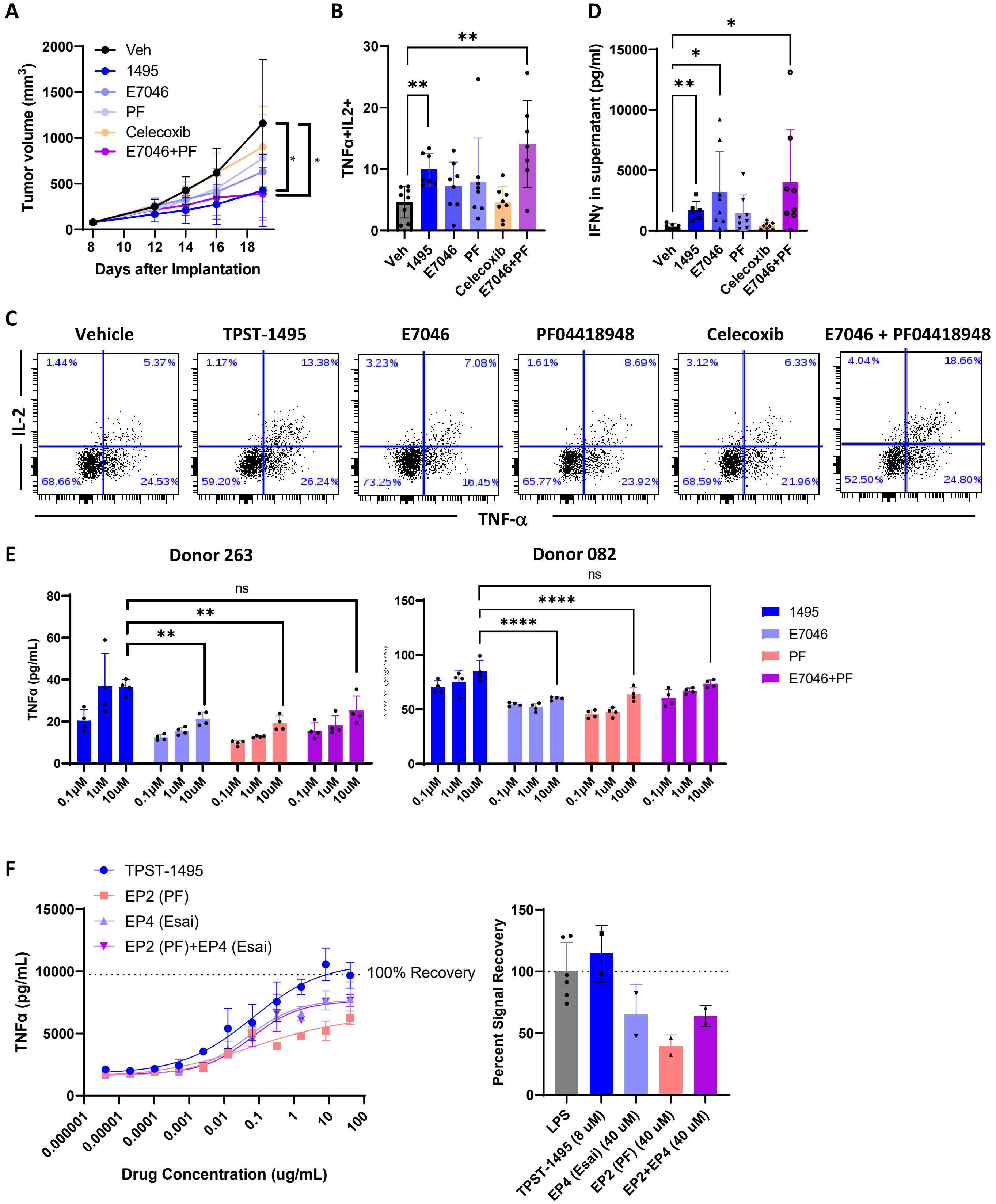

A
B
D
Veh
1495
E7046
PF
Celecoxib
E7046+PF
Tumor volume (mm³)
Days after Implantation
TNFα+IL2+
IFNγ in supernatant (pg/ml)
C
Vehicle
TPST-1495
E7046
PF04418948
Celecoxib
E7046 + PF04418948
IL-2
TNF-α
E
Donor 263
Donor 082
TNFα (pg/mL)
ns
0.1uM
1uM
10uM
F
TPST-1495
EP2 (PF)
EP4 (Esai)
EP2 (PF)+EP4 (Esai)
TNFα (pg/mL)
100% Recovery
Drug Concentration (ug/mL)
Percent Signal Recovery
LPS
TPST-1495 (8 uM)
EP4 (Esai) (40 uM)
EP2 (PF) (40 uM)
EP2+EP4 (40 uM)

Figure S3: Combination of Single EP2 and EP4 antagonism mirrors dual antagonism by TPST-1495

A) Tumor outgrowth of EG7 tumors in mice treated for 10 days with 100mg/kg BID TPST-1495, 150mg/kg QD E7046, 100mg/kg QD PF04418948, or a combination of E7046 and PF04418948 at the previously listed regimens.

B,C) ICS (B) and example flow cytometry plots (C) from TILs in (A) after ex vivo T cell stimulation. Tumors were processed to single cell suspension, then stimulated with PMA/Ionomycin in the presence of Golgi stop and Golgi plug, then stained for cytokine production and analyzed by flow cytometry.

D) Cytokine measurement from supernatants of ex vivo T cell stimulation from TILs as in (A-C). Cells were isolated and treated as in (B,C), but in the absence of Golgi stop and Golgi plug.

E) IFN-γ concentration in supernatants of PGE2 blockade assay performed on magnetically enriched T Cells from human PBMC. Cells were treated with labelled concentrations of TPST-1495, E7046, PF04418948 (PF), or both, followed by 333nM PGE2, then CEF peptides before supernatants were analyzed via bead-based cytokine array analysis. Graphs show data with N=4 technical replicates from one of 1 experiment using 2 different donors.

F) TNFα concentrations and quantification of percent recovery were measured from whole blood as described in Figure 2A. Graphs show data that is representative of N=2 technical replicates from 1 experiment.

Mean and Standard Deviation are depicted in all graphs and the Student's two-sided t-test was used to assess significance between individual groups. ****, $p<0.0001$. ***, $p<0.001$. **, $p<0.01$. *, $p<0.05$.

Figure S4

A

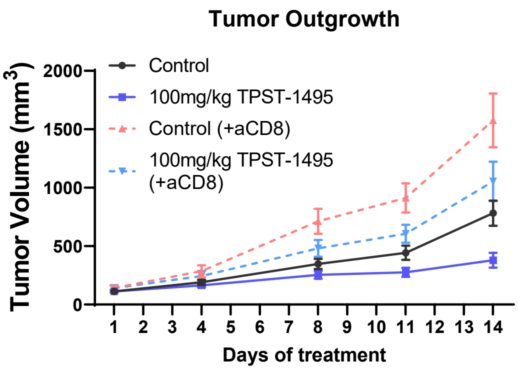

Group	Tumor average	Tumor Growth inhibition
Control	782.13	
TPST-1495	379.20	51.52
Control+aCD8	1575.78	
TPST-1495+aCD8	1054.54	33.08

B

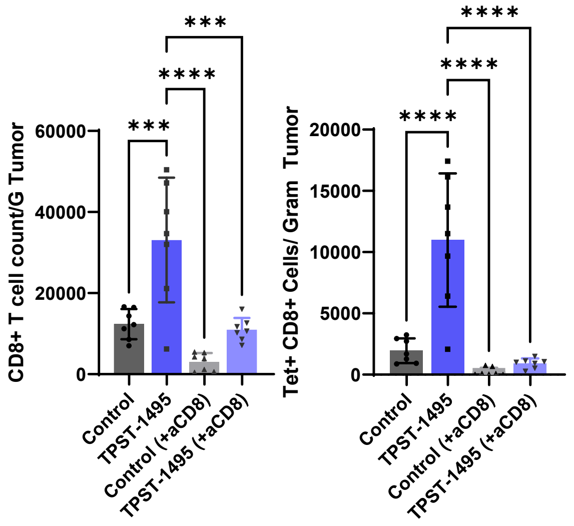

C

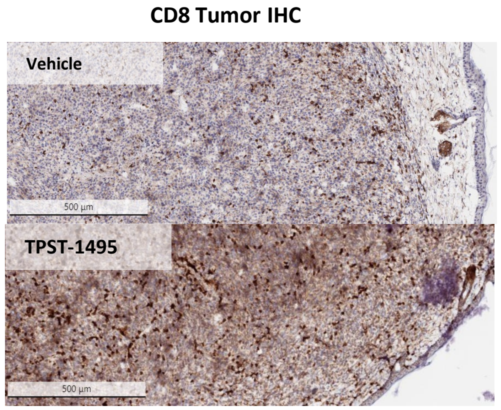

Figure S4: TPST-1495 Displays T cell-independent anti-tumor efficacy in CT26.

A) Tumor outgrowth of CT26 tumors in mice treated for 14 days with 100mg/kg TPST-1495 and/or anti-CD8α depleting antibody. Table below depicts tumor averages and calculated TGI where TGI=100*(Control Volume- Treatment Volume)/Control Volume.

B) Flow cytometry counts of tumor infiltrating lymphocyte populations normalized to tumor mass from experiment in (A). Data are representative of 1 experiment with 7 animals. Mean and standard deviation are depicted in all graphs and the ordinary one-way ANOVA with Tukey's multiple comparisons test was used to assess significance between individual groups. ****, $p<0.0001$. ***, $p<0.001$. *, $p<0.05$.

C) Example of CD8α -targeted immunohistochemistry performed on tumors from A,B.

Figure S5

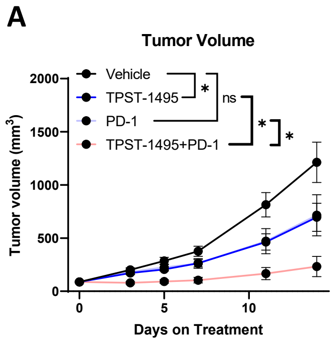

Figure S5: Increased efficacy with TPST-1495 and α-PD-1 combination therapy in CT26 model.

A) Tumor outgrowth in BALB/c mice implanted with CT26 cells and treated with 100mg/kg BID PO TPST-1495 +/- 200mg Q3D IP ant-PD-1 antibody. Results are representative of 2 experiments with at least 5 mice.

Figure S6

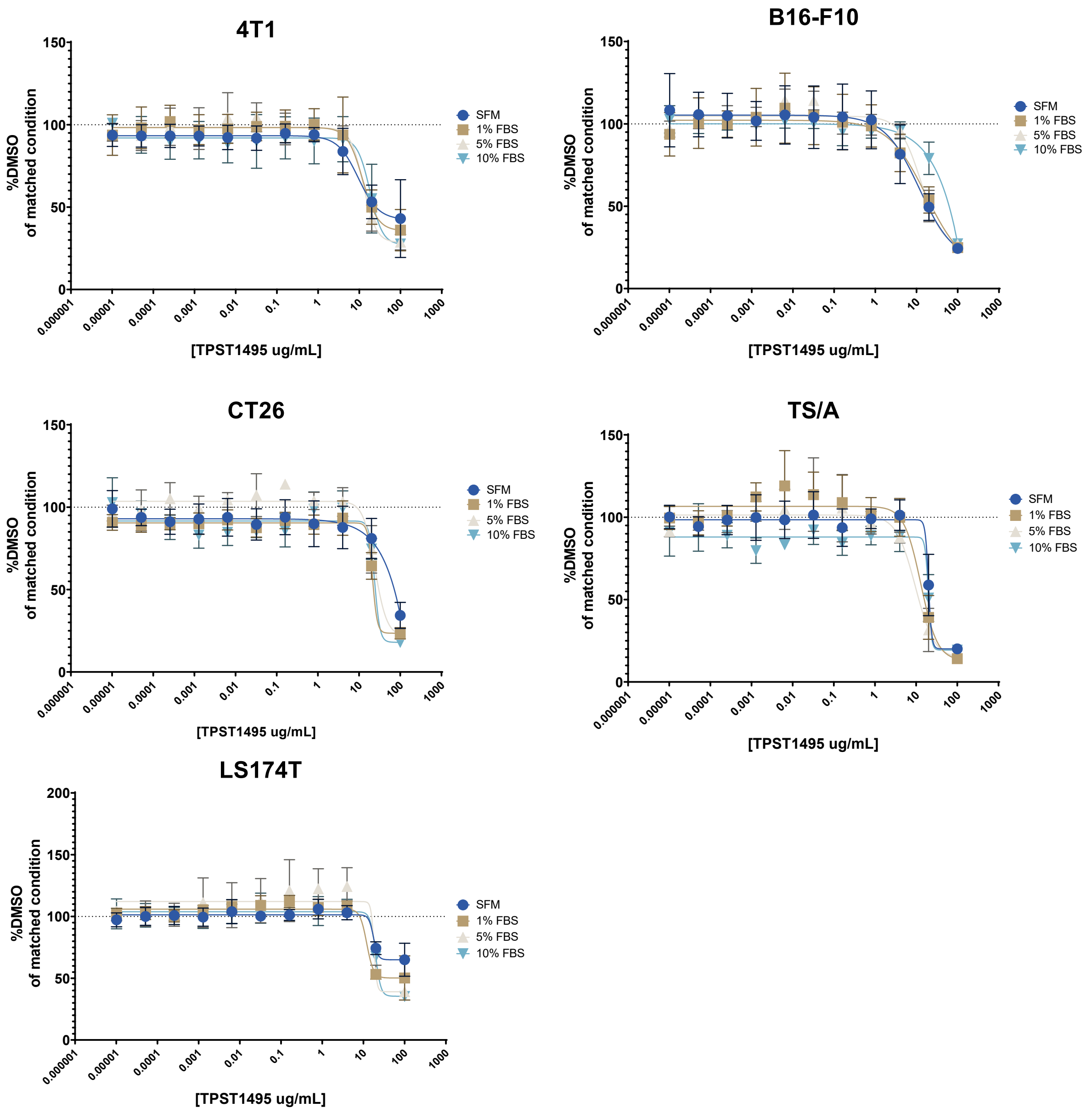

Figure S6: TPST-1495 displays little tumor cell cytotoxicity in vitro at pharmacologically relevant concentrations

A) MTT assay results displayed as percent viability as compared to DMSO normalized control wells. Cells were plated the previous day and allowed to adhere overnight, then subjected to increasing amounts of TPST-1495 in labelled percent serum conditions at 0 hours and analyzed by MTT assay 48 hours later.

Figure S7

A

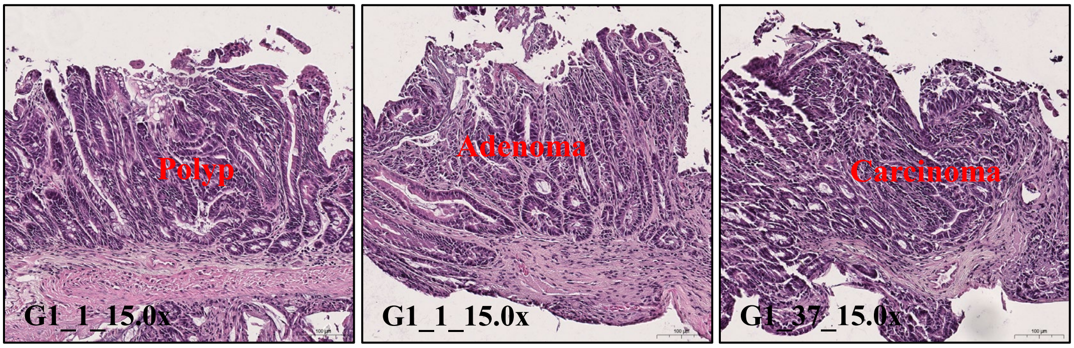

B

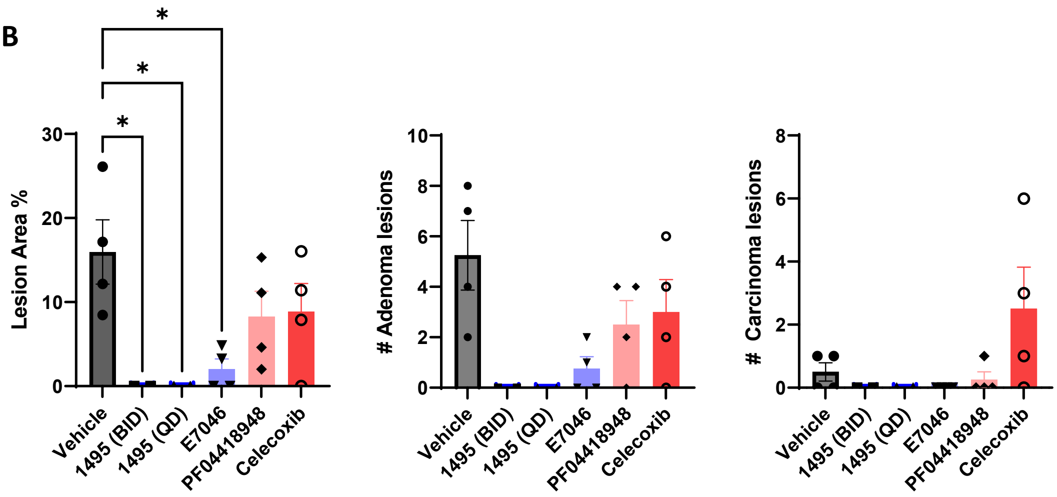

Figure S7: Histopathological analysis of resected APC$^{min/+}$ colon hyperplasia

A) Histology of areas of hyperplasia resected from the vehicle treated group in the experiment in Figure 5B. Blinded independent analysis identified 3 distinct degrees of pathologies in small intestines of mice.

B) Quantification of blinded histopathological analysis from experiment in Figure 5B. Mean and standard deviation are depicted in all graphs and the ordinary one-way ANOVA with Tukey's multiple comparisons test was used to assess significance between individual groups. *, $p<0.05$.

Figure S8

A

TPST-1495 Vs Vehicle

Gene	Log2 Fold Change
Grik3	2.10
Fendrr	1.93
Gm43621	1.90
Exoc3l2	1.47
Hlf	1.41
Papln	1.11
Smim3	-1.10
Bace2	-1.17
Pde10a	-1.61
Ggh	-1.74
Slc38a4	-2.32
Ripply1	-2.38
Nptx2	-2.69
Scgb2b20	-3.19
Gkn1	-3.91

E7046 Vs Vehicle

Gene	Log2 Fold Change
Gm15609	2.15
Hlf	1.60
Fendrr	1.44
Gria4	1.44
Gm43621	1.40
Dbp	1.31
Ablim2	1.21
Ciart	1.14
Exoc3l2	1.06
Ggh	-1.20
Reg4	-1.44
Bpifb1	-1.80
Nptx2	-2.16
Scgb2b20	-2.33
Gkn1	-2.62

PF04418948 Vs Vehicle

Gene	Log2 Fold Change
Hlf	1.83
Dbp	1.75
Mamdc2	1.58
Sypl2	1.39
Ciart	1.39
Podn	1.30
Per2	1.26
Per3	1.15
Tef	1.08
Gm43585	-1.79
Snca	-2.60
Hbq1b	-2.74
Hbb-bs	-3.28
Alas2	-3.48
Slc4a1	-4.11

Celecoxib Vs Vehicle

Gene	Log2 Fold Change
Mamdc2	2.18
Hlf	2.03
Grik3	1.80
Gm43621	1.74
Dbp	1.72
Fendrr	1.55
Per1	1.51
Ciart	1.35
Tns2	1.13
Lipg	1.01
Anxa1	-1.02
Tcf23	-1.39
Gm10371	-1.55
1500009C09Rik	-2.41
1700045H11Rik	-2.69

B

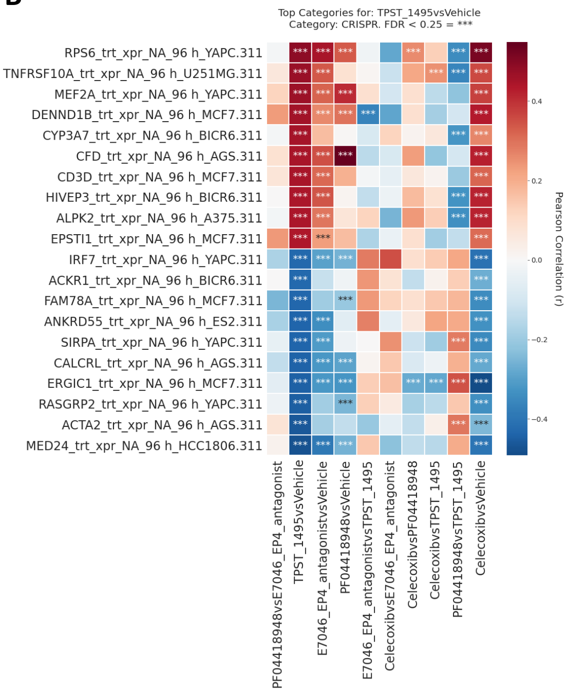

Figure S8: Co-regulated genes determined from RNA sequencing of hyperplasia in APC$^{min/+}$ mice treated with prostaglandin pathway targeted agents

A) Top 15 up- and down-regulated significant genes from RNA sequencing of hyperplasia pulled from APC$^{min/+}$ small intestines represented as a heat map. Significance was defined as p<0.05 and FDR<0.25 as compared to vehicle treated animals. Genes that are shared between at least one other group are in bold.

B) Comparison of the RNA sequencing data from tumors in (A) to the L1000[46] database depicted as a heat map with red representing positive Pearson correlation and blue representing a negative Pearson correlation.
